# Supplementary material for: Gram-scale selective telomerization of isoprene and CO2 toward 100% renewable materials
Source: Nat Commun. 2025 Aug 28;16:7326. doi: 10.1038/s41467-025-62409-2 (PMC12394455; doi:10.1038/s41467-025-62409-2)
Supplement: Supplementary file 1 — Supplementary Information [file 41467_2025_62409_MOESM1_ESM.pdf]

*Supplementary Information for*

# *Gram-Scale Selective Telomerization of Isoprene and $CO_2$ Toward 100% Renewable Materials*

Marius D. R. Lutz, Felix Kracht, Kota Marumoto and Kyoko Nozaki*

*Department of Chemistry and Biotechnology, Graduate School of Engineering, The University of Tokyo, 7-3-1 Hongo, Bunkyo-ku, Tokyo 113-8656, Japan*
**Email: nozaki@chembio.t.u-tokyo.ac.jp*

# Contents

**1. Supplementary Methods .......... 3**

1.1 General information .......... 3

1.2 Preparation of lactone 1 .......... 3

1.3 Polymerization of 1 .......... 4

1.4 Synthesis of other reagents .......... 5

1.5 Optimization of Telomerization of Isoprene and $CO_2$ .......... 6

1.6 Optimization of Polymerization of Lactone 1 .......... 17

**2. Supplementary Discussion .......... 21**

2.1 Computational Details .......... 21

2.2 Discussion of the free energy diagram of the reaction .......... 22

2.3 Benchmarking .......... 22

2.4 Summary of Calculated Energy Values .......... 23

**3. Supplementary Notes .......... 25**

3.1 NMR spectra .......... 25

3.2 IR spectra .......... 28

3.3 GC-MS spectra ..... 30

3.4 SEC, TG and DSC charts ..... 31

**4. Supplementary References ..... 32**

# 1. Supplementary Methods

## 1.1 General information

### General

All reactions and polymerizations were carried out using a standard argon-filled glovebox or Schlenk techniques under nitrogen. Lactone synthesis was performed in a 50 mL stainless autoclave.

### Material

Carbon dioxide (>99.990 vol%) and ethylene (>99.9%) were purchased from Takachiho Chemical Industrial Co. (Takachiho) and used after passing through a dry column DC-HDF300-A3 made by Nikka Seiko Co., Ltd. Anhydrous acetonitrile, palladium(II) acetylacetonate ($Pd(acac)_2$), tris(*p*-tolyl)phosphine ($P(p\text{-Tol})_3$) were purchased from Kanto Chemical Co. Inc. (Kanto), Wako Pure Chemical Industries, Ltd., Wako Pure Chemical Industries, Ltd., respectively, and used as received. Isoprene was purchased from Tokyo Chemical Industry Co. Ltd. (TCI), distilled from calcium hydride, degassed by freeze-pump-thawing and stored over activated molecular sieves before use. Tetrabutylammonium acetate (TBAAc) (97%) was purchased from Sigma Aldrich and used as received and stored under an inert atmosphere. The other reagents were purchased from TCI, Kanto, Sigma-Aldrich Chemical Co., or Wako Pure Chemical Industries, Ltd. and were used as received.

### Instrumentation

**NMR spectra** were recorded on 400 and 500 MHz spectrometers (JEOL JNM-ECS400 or BRUKER Ascend500) at ambient temperature unless otherwise noted. Chemical shift values for protons are referenced to the residual proton resonance of chloroform-*d* (δ: 7.26). Chemical shift values for carbons are referenced to the carbon resonances of chloroform-*d* (δ: 77.0).
**Gas chromatography:** Yield of lactone **1** was determined by Shimadzu GC-2014 equipped with InertCap 5MS/Sil capillary column (0.25 ID, 0.25 μm df 30 m) with a calibration curve using n-decane as internal standard. The selectivity of lactone **1** was determined based on the area ratio of **1a** and **1b** to all other products arising from isoprene.
**Infrared (IR)** spectra were recorded on a Shimadzu FTIR-8400 equipped with an attenuated total reflection (ATR) system.
**Size-exclusion chromatography (SEC)** analyses were carried out with two columns (Shodex KF-804L) using chloroform ($CHCl_3$) as an eluent at 40 °C at 1 mL/min. The molecular weight was calibrated against standard polystyrene samples.
**Differential scanning calorimetry (DSC)** measurements of polymers were performed on a Seiko DSC 7020 analyzer at a heating and cooling rate of 20 °C/min. Glass-transition temperature (Tg) values were determined from the third heating scan.
**Thermogravimetric (TG)** analyses were performed on a Seiko EXSTAR 6000 TG/DTA 6200 analyzer at a heating rate of 10 °C/min under nitrogen.

## 1.2 Preparation of lactone 1

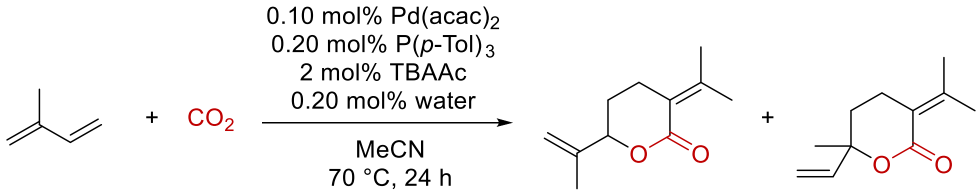


**1a** **1b**

A 50 mL stainless steel autoclave equipped with a magnetic stirring bar was removed from a 120 °C oven, charged with Pd(acac)$_2$ (38.1 mg, 0.125 mmol, 0.10 mol%), and P(*p*-Tol)$_3$ (76.1 mg, 0.250 mmol, 0.20 mol%) and introduced into a Glovebox. TBAAc (753.8 mg, 2.500 mmol, 2.0 mol%) was added, and the autoclave was quickly closed under ambient atmosphere, evacuated, and back-filled with nitrogen three times. Then, acetonitrile (11.5 mL), water (1.0 mL, 0.20 mol%, 0.25 M in MeCN), and isoprene (8.41 g, 12.50 mL, 125.0 mmol, 1 equiv.) were charged through the autoclave head via syringe, and the autoclave was sealed. The autoclave was pressurized with 4.0 MPa of $CO_2$ for 5 minutes under stirring. After stirring at 70 °C for 24 h, the autoclave was cooled to room temperature with a water bath. After residual gas pressure was released, the volatile matters were removed by evaporation. Conversion and selectivity were determined by subjecting an aliquot to GC-FID analysis using n-decane as external standard. The obtained oil was purified by column chromatography on silica gel (gradient, 2 to 20% ethyl acetate/hexane). The obtained light-yellow oil was further purified by vacuum distillation (180 °C) to afford **1a** and **1b** as an inseparable mixture; yield: 1.63 g (15%).

Note: The reaction is sensitive to the amount of water in the system and the ambient conditions. Over the course of six months we found that the optimal amount of water fluctuates between 0.2 and 0.5 mol%.

$^1$H NMR (500 MHz, $CDCl_3$) $\delta$ Isomer **1a**: 5.04 (s, 1H), 4.94 (s, 1H), 4.56 – 4.49 (m, 1H), 2.53 – 2.47 (m, 2H), 2.20 (s, 2H), 2.03 – 1.97 (m, 1H), 1.94 (dd, $J$ = 6.1, 4.9 Hz, 1H), 1.84 (s, 4H), 1.79 (s, 3H); Isomer **1b**: 5.82 (dd, $J$ = 17.3, 10.9 Hz, 1H), 5.18 (d, $J$ = 17.3 Hz, 1H), 5.11 (d, $J$ = 10.9 Hz, 1H), 2.46 – 2.39 (m, 2H), 2.22 (s, 3H), 1.91 (dd, $J$ = 5.4, 5.4 Hz, 1H), 1.82 (s, 4H), 1.42 (s, 3H).

$^{13}$C NMR (126 MHz, $CDCl_3$) $\delta$ Isomer **1a**: 166.9, 151.6, 142.6, 120.0, 113.0, 81.1, 27.4, 25.1, 23.8, 23.2, 18.4; Isomer **1b**: 166.4, 152.1, 140.5, 119.3, 114.4, 81.5, 32.7, 27.7, 24.0, 23.5, 23.3.

IR (ATR): 752, 924, 961, 984, 1018, 1072, 1090, 1115, 1180, 1206, 1225, 1283, 1373, 1412, 1439, 1618, 1705 (s), 2342, 2361, 2930, 2980 cm$^{-1}$

Anal. calcd for $C_{11}H_{16}O_2$: C, 73.30; H, 8.95; N, 0. Found: C, 73.52; H, 8.68; N, 0.

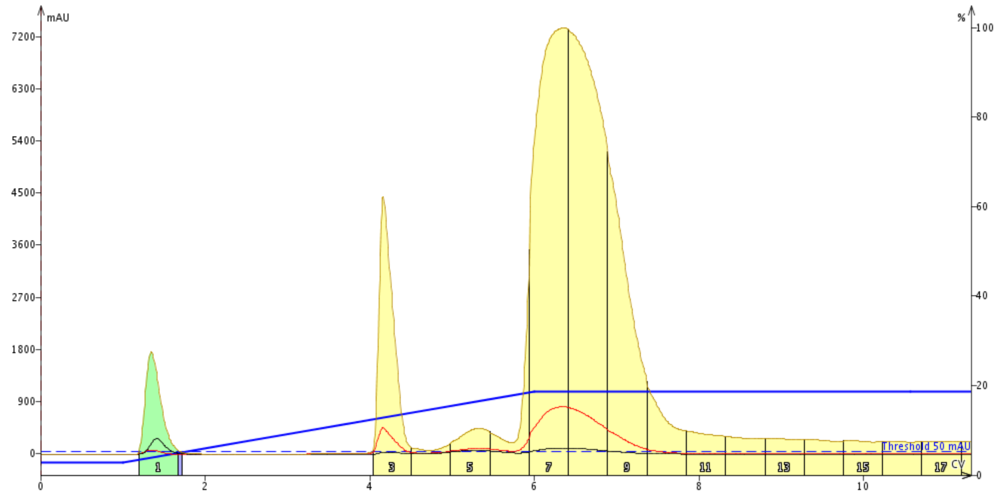


**Figure S1**. Chromatogram of column chromatography purification of the product. The product eluted in fractions 7-10.

## 1.3 Polymerization of 1

A mixture of lactone **1** (0.50 mmol), $ZnCl_2$ (0.50 mmol), and ethylene carbonate (2.0 mmol) was stirred at 120 ºC for 24 hours in a 5 mL vial under an argon atmosphere. Upon cooling, an aliquot was analyzed by $^1$H NMR spectroscopy to determine the

conversion of **1**. The resulting solid mixture was diluted THF (0.5 mL) and precipitated into an excess amount of methanol (15 mL). The formed precipitate was collected and washed with methanol (2x5 mL). The remaining solid was dissolved in THF (1 mL) and reprecipitated with methanol (10 mL) to afford **2**; yield: 7.5 mg (8%); $M_n$ = 3.88 kg/mol, $M_w$ / $M_n$ = 1.28 ($CHCl_3$, RALS); $^1H$ NMR, $^{13}C$ NMR, IR spectra and their assignments are described in pages S25–S29. The SEC, TG, and DSC charts are shown on page S31.

# 1.4 Synthesis of other reagents

## 1.4.1 Preparation of colloidal nanoparticles

Colloidal Pd nanoparticles were synthesized according to a modified procedure by Reetz et al.[1] A 80 mL Schlenk tube was charged with $Pd(OAc)_2$ (0.15 mmol) and THF (50 mL) and heated to 60 °C. To the light orange solution was added a solution of TBAAc (0.50 mmol) in THF (5.0 mL) via syringe. The reaction was stirred for 4 hours at this temperature, during which the color of the solution changed to light yellow and then darkened. The volatiles were removed under reduced pressure to obtain Pd nanoparticles. The particles were redispersed in 15.0 mL MeCN (10.0 mM) to afford a black dispersion.

## 1.4.2 Synthesis of TBAAc

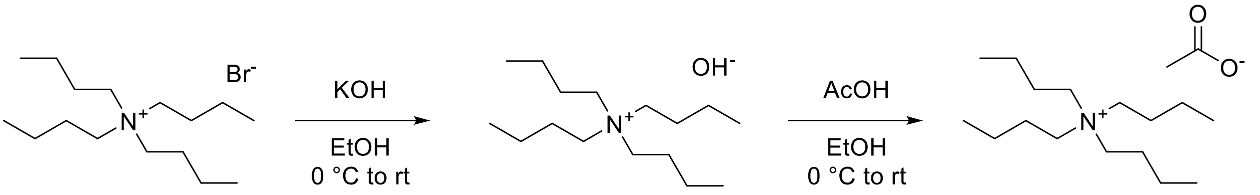


10.0 g

quant.

Tetrabutylammonium acetate (TBAAc) was synthesized according to a modified literature procedure.[2,3] Anhydrous tetrabutylammonium bromide (10.0 g, 31.0 mmol, 1.0 equiv.) was dissolved in ethanol (20 mL) in a 250 mL round-bottomed flask equipped with a magnetic stirring bar under ambient conditions. When a clear solution was obtained, the mixture was cooled to 0 °C before adding a solution of KOH (1.91 g, 34.1 mmol, 1.1 equiv.) in ethanol (15 mL) in portions. A white suspension formed and the mixture was stirred at 0 °C for 15 min and at rt for 15 min. The precipitated KBr was filtered off, and the filtrate was cooled to 0 °C. A solution of acetic acid (2.05 g, 34.1 mmol, 1.1 equiv.) in ethanol (5 mL) was added dropwise while monitoring the pH of the solution. When the pH reached 6, addition was stopped and the resulting slightly viscous solution was stirred at rt for 30 min. The volatiles were removed at a rotary evaporator (water bath temperature 60 °C), and the resulting solid was collected, crushed, and dried in a vacuum oven at 60 °C until constant weight was reached and $^1H$ NMR and FTIR showed no remaining water signal. The analytical data is in accordance with the literature.[2,3]

$^1H$ NMR (400 MHz, $CDCl_3$) $\delta$ 3.47 – 3.33 (m, 8H), 1.97 (s, 3H), 1.74 – 1.61 (m, 8H), 1.52 – 1.38 (m, 8H), 1.01 (t, J = 7.3 Hz, 12H).
IR (ATR): 2958, 2873, 1581, 1495, 1466, 1371, 1054, 1030, 883, 739, 635 $cm^{-1}$

## 1.5 Optimization of Telomerization of Isoprene and $CO_2$

### 1.5.1 Initial optimization

**General procedure:**

A 50 mL stainless steel autoclave equipped with a magnetic stirring bar was removed from a 120 °C oven, charged with $Pd(acac)_2$ (7.6 mg, 0.025 mmol, 0.10 mol%), and $P(p\text{-Tol})_3$ (15.2 mg, 0.050 mmol, 0.20 mol%) and introduced into a Glovebox. TBAAc (150.8 mg, 0.500 mmol, 2.0 mol%) was added, and the autoclave was quickly closed under an ambient atmosphere, evacuated, and backfilled with nitrogen three times. Then, acetonitrile (2.30 mL), water (0.20 mL, 0.25 M in MeCN), and isoprene (1.70 g, 2.50 mL, 25.0 mmol, 1 equiv.) were charged through the autoclave head via syringe, and the autoclave was sealed. The autoclave was pressurized with 4.0 MPa of $CO_2$ for 5 minutes under stirring. After stirring at 70 °C for 24 h, the autoclave was cooled to room temperature with a water bath. After residual gas pressure was released, the volatile matters were removed by evaporation. Conversion and selectivity were determined by subjecting an aliquot to GC-FID analysis using n-decane as external standard.

**Initial screening:**

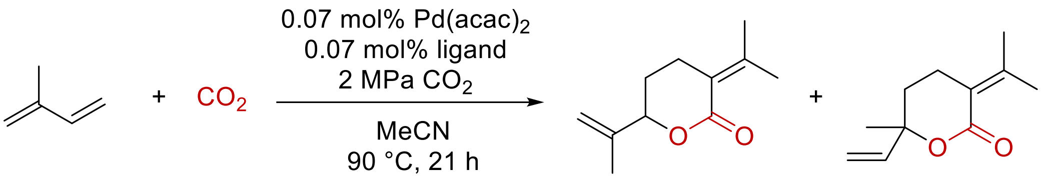


**Table S1:** Initial screening of ligands.

| Entry[a] | [Pd] (mol%) | Ligand | Main Product | Side Product | Mass of non-volatiles |
|---|---|---|---|---|---|
| 1 | 0.07 | $[Fe(Cp\{P^iPr_2\})_2]$ | Higher Terpenes (M ≥ 329 g/mol) | Terpenes | 59.6 mg |
| 2 | 0.07 | $N^iPr_2Et$ | Terpenes | Sesquiterpenes, Lactones (Traces) | 28.7 mg |
| 3 | 0.07 | $Me_2N(CH_2)_2NMe_2$ | Lactone, Terpenes | Terpenes, Sesquiterpenes, Furans | 58.7 mg |
| 4 | 0.07 | $Et_2N(CH_2)_2NEt_2$ | Lactone | Terpenes, Sesquiterpenes | 41.5 mg |
| 5 | 0.07 | $Me_2N(CH_2)_4NMe_2$ | Sesquiterpenes | Terpenes | 15.6 mg |
| 6 | 0.07 | *N*-Me -Piperazine | Sesquiterpenes | Terpenes | 6.5 mg |
| 7 | 0.07 | 3,5-Dimethylpyrazole | No conversion | No Conversion | 5.2 mg |
| 8[b] | 0.05 | TBAAc | Lactone | Terpenes, Sesquiterpenes | 10.1 mg |

[a] Reaction conditions: isoprene (48.3 mmol), $Pd(acac)_2$ (0.07 mol%), ligand (0.07 mol%), MeCN (2.5 mL), $CO_2$ (2 MPa), 90 °C, 21 h. [b] 0.05 mol% $Pd(acac)_2$, 1 mol% TBAAc, 70 °C.

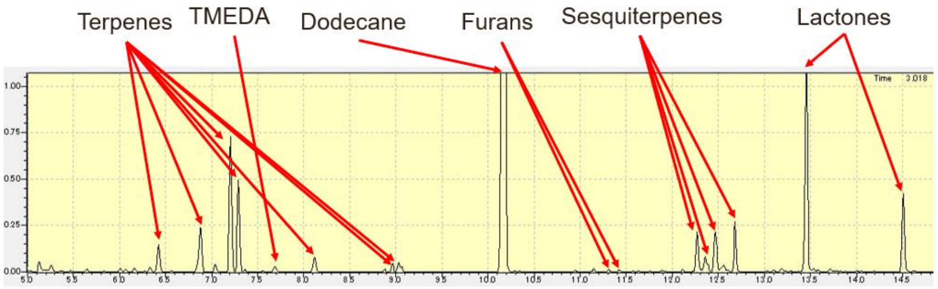


**Figure S2**. Assigned GC-MS chromatogram of Table S1, entry 3.

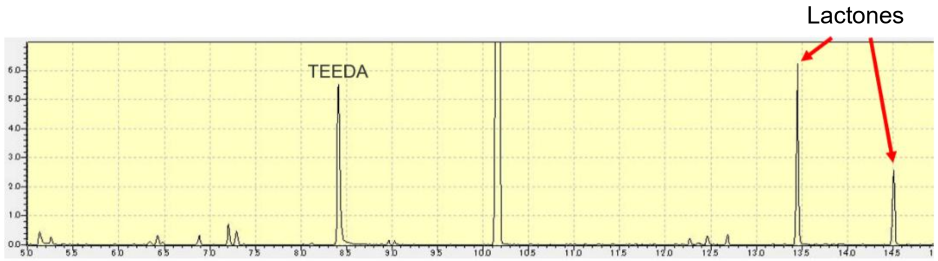


**Figure S3**. Assigned GC-MS chromatogram of Table S1, entry 4.

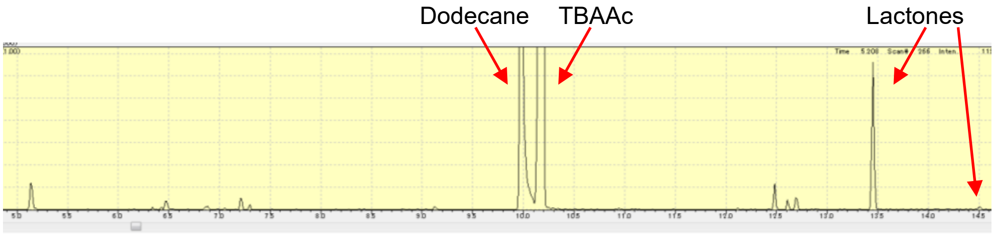


**Figure S4**. Assigned GC-MS chromatogram of Table S1, entry 8.

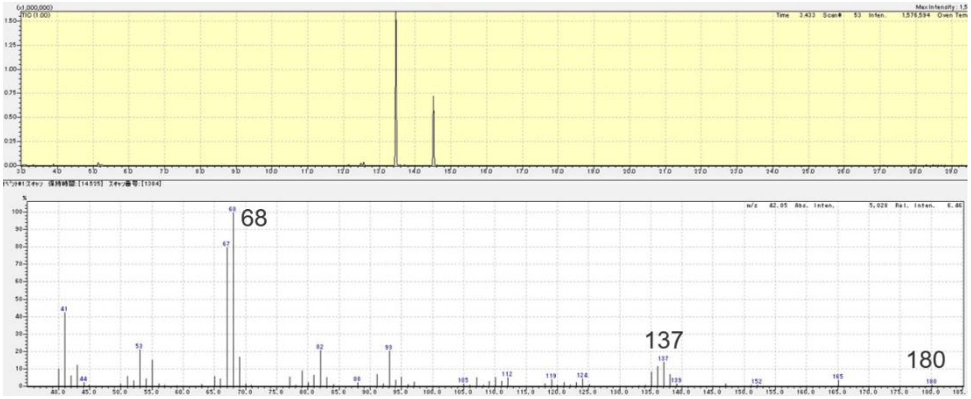


**Figure S5**. Assigned GC-MS chromatogram of isolated lactone mixture.

**Table S2:** Screening of catalyst loading, time, and temperature with TBAAc.

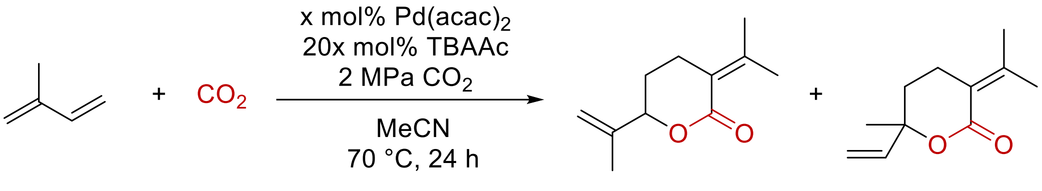


**1a** **1b**

| Entry[a] | [Pd] (mol%) | Deviation from above | Yield **1a** + **1b**[b] | Ratio **1a** / **1b** | Selectivity (%)[c] | TON[d] |
|---|---|---|---|---|---|---|
| 1 | 0.05 | None | 0.2% | 1 : 12 | 49 | 4 |
| 2 | 0.26 | None | 1.4% | 1 : 12 | 47 | 5 |
| 3 | 0.53 | None | 2% | 1 : 12 | 69 | 4 |
| 4 | 0.26 | 3 days | 5% | 1 : 11 | n.d. | 19 |

[a] Reaction conditions: isoprene (50 mmol), Pd(acac)$_2$ (x mol%), TBAAc (20x mol%), MeCN (5 mL), $CO_2$ (2 MPa), 70 °C, 24 h. [b] isolated yield after pTLC purification. [c] Determined by GC-FID area ratio of **1** versus all isoprene-derived products. [d] n(**1a**+**1b**)/n(Pd).

**Reaction optimization:**

**Table S3:** Screening of ligands.

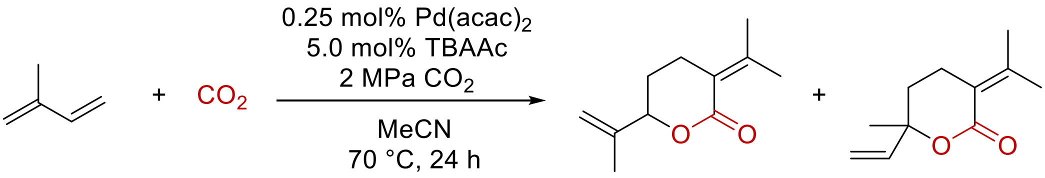


**1a** **1b**

| Entry[a] | Deviation from above | Yield **1a** + **1b**[b] | Ratio **1a** / **1b** | Selectivity (%)[c] | TON[d] |
|---|---|---|---|---|---|
| 1 | None | 2% | 1 : 21 | 11 | 4 |
| 2 | 2.5 M (10 mL MeCN) | 2% | 1 : 10 | 14 | 4 |
| 3 | 0.05 mol% Pd(acac)$_2$, 1.0 mol% TBAAc | 1.3% | 1 : 9 | 28 | 3 |
| 4 | TEEDA (0.25 mol%) instead of TBAAc | <1% | n.d. | n.d. | n.d. |
| 5 | dcypb (0.5 mol%) instead of TBAAc | <1% | n.d. | n.d. | n.d. |
| 6 | $PPh_3$ (0.5 mol%) | 4% | 1 : 12 | 28 | 8 |
| 7 | P(*o*-OH-Ph)$Ph_2$ (0.5 mol%) instead of TBAAc | 0 | n.d. | n.d. | n.d. |
| 8 | P(*p*-OMe-Ph)$_3$ (0.5 mol%) instead of TBAAc | <1% | n.d. | n.d. | n.d. |

[a] Reaction conditions: isoprene (25 mmol), Pd(acac)$_2$ (0.25 mol%), ligand (0.50 mol%), TBAAc (5.0 mol%), MeCN (2.5 mL), $CO_2$ (2 MPa), 70 °C, 24 h. [b] GC-FID yield with external standard *n*-decane. [c] Determined by GC-FID area ratio of **1** versus all isoprene-derived products. [d] n(**1a**+**1b**)/n(Pd).

**Table S4:** Screening of catalyst loading.

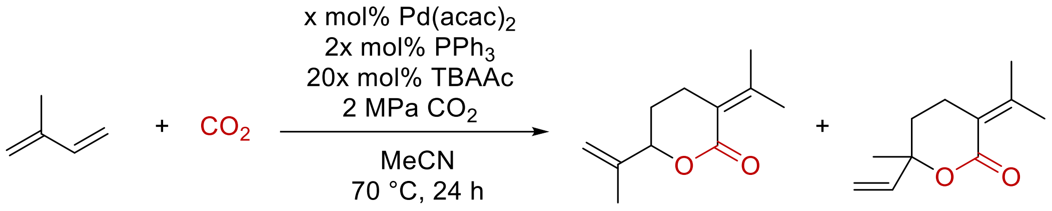


**1a** **1b**

| Entry[a] | [Pd] (mol%) | Yield **1a** + **1b**[b] | Ratio **1a** / **1b** | Selectivity (%)[c] | TON [d] |
|---|---|---|---|---|---|
| 1 | 0.25 | 4% | 1 : 12 | 9 | 8 |
| 2 | 0.10 | 8% | 1 : 1.8 | 82 | 38 |
| 3 | 0.05 | 1.7% | 1 : 5 | 68 | 17 |
| 4 | 0.05 (at 80 °C) | 0.7% | 1 : 3 | 47 | 7 |

[a] Reaction conditions: isoprene (25 mmol), Pd(acac)$_2$ (x mol%), P(*p*-Tol)$_3$ (2x mol%), TBAAc (20x mol%), MeCN (2.5 mL), $CO_2$ (2 MPa), 70 °C, 24 h. [b] GC-FID yield with external standard *n*-decane. [c] Determined by GC-FID area ratio of **1** versus all isoprene-derived products. [d] n(**1a**+**1b**)/n(Pd).

**Table S5:** Screening of ligands.

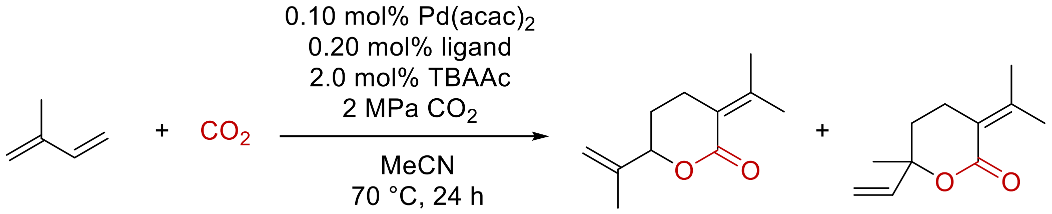


**1a** **1b**

| Entry[a] | Ligand | Yield **1a** + **1b**[b] | Ratio **1a** / **1b** | Selectivity (%)[c] | TON [d] |
|---|---|---|---|---|---|
| 1 | PPh$_3$ | 12% | 1 : 3 | 69 | 62 |
| 2 | PPh$_3$ + *p*-hydroquinone (0.4 mol%) + NEt$_3$ (2.0 mol%) | 6% | 1 : 4 | 60 | 29 |
| 3 | P(*o*-Tol)$_3$ | 8 | 1 : 1.8 | 70 | 38 |
| 4 | P(*o*-OMe-Ph)$_3$ | 1.9 | 1 : 10 | 30 | 10 |
| 5 | P(*o*-OH-Ph)Ph$_2$ | 7 | 1 : 5 | 55 | 36 |
| 6 | P(*p*-Tol)$_3$ | 17 | 1 : 2.0 | 78 | 82 |
| 7 | 2-(*o*-Tol)-1-P(Cy)$_2$-Ph (“*o*-Tol-Cy-Johnphos”) | 1.0 | 1 : 11 | 31 | 5 |

| | | | | | |
|---|---|---|---|---|---|
| 8 | XPhos | 0.3 | 1 : 1.6 | 4 | 1 |
| 9 | $PCy_3$ | 1.3 | 1 : 7 | 60 | 6 |
| 10 | dcypb | 0.2 | 1 : 1.3 | 5 | 1 |
| 11 | dppb | 0.7 | 1 : 2.0 | 9 | 4 |
| 12 | IMes (* HCl + cat. KOtBu) | 0 | n.d. | n.d. | n.d. |
| 13 | IPr-PEPPSI (instead of $Pd(acac)_2$) | 0 | n.d. | n.d. | n.d. |

[a] Reaction conditions: isoprene (25 mmol), $Pd(acac)_2$ (0.10 mol%), ligand (0.20 mol%), TBAAc (2.0 mol%), MeCN (2.5 mL), $CO_2$ (2 MPa), 70 °C, 24 h. [b] GC-FID yield with external standard *n*-decane. [c] Determined by GC-FID area ratio of **1** versus all isoprene-derived products. [d] n(**1a**+**1b**)/n(Pd).

**Table S6:** Screening of palladium source.

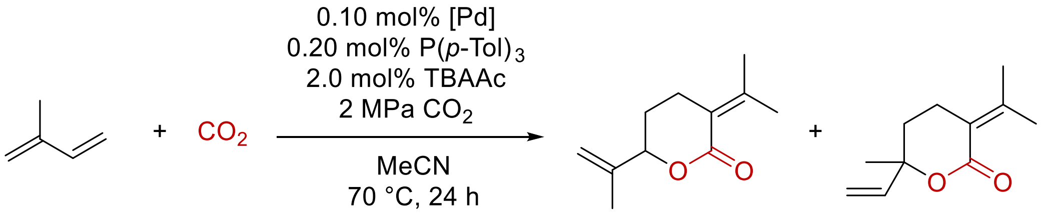


**1a** **1b**

| Entry[a] | Pd source | Yield **1a** + **1b**[b] | Ratio **1a** / **1b** | Selectivity (%)[c] | TON[d] |
|---|---|---|---|---|---|
| 1 | $Pd(acac)_2$ | 17% | 1 : 2.0 | 78 | 82 |
| 2 | $Pd(OAc)_2$ | 11% | 1 : 4 | 56 | 55 |
| 3 | $Pd(COD)Cl_2$ | 8% | 1 : 2.2 | 78 | 40 |
| 4 | $Pd(MeCN)_2Cl_2$ | 8% | 1 : 2.4 | 61 | 40 |
| 5 | $[Pd(allyl)Cl]_2$ | <1% | 1 : 1.1 | 1 | 1 |
| 6 | $Pd_2(dba)_3 \cdot CHCl_3$ | <1% | 1 : 0.6 | 7 | 1 |
| 7 | “Pd EnCat 30” ($Pd(OAc)_2$ @ polyurea matrix) | <1% | 1 : 7 | 40 | 1 |
| 8 | Pd NPs, w/out $P(pTol)_3$ nor TBAAc | 0 | n.d. | n.d. | n.d. |
| 9 | Pd NPs + $P(pTol)_3$, w/out TBAAc | 0 | n.d. | n.d. | n.d. |
| 10 | Pd NPs + TBAAc, w/out $P(pTol)_3$ | 0 | n.d. | n.d. | n.d. |
| 11 | Pd NPs + $P(pTol)_3$ + TBAAc | 0 | n.d. | n.d. | n.d. |
| 12 | $Pd/CeO_2$[e] | 0 | n.d. | n.d. | n.d. |

[a] Reaction conditions: isoprene (25 mmol), [Pd] (0.10 mol%), $P(p\text{-}Tol)_3$ (0.20 mol%), TBAAc (2.0 mol%), MeCN (2.5 mL), $CO_2$ (2 MPa), 70 °C, 24 h. [b] GC-FID yield with external standard *n*-decane. [c] Determined by GC-FID area ratio of **1** versus

all isoprene-derived products. [d] n(**1a**+**1b**)/n(Pd). [e] Pretreated under $H_2$ atmosphere at 200 °C for 1 h.

**Table S7:** Screening of reaction temperature.

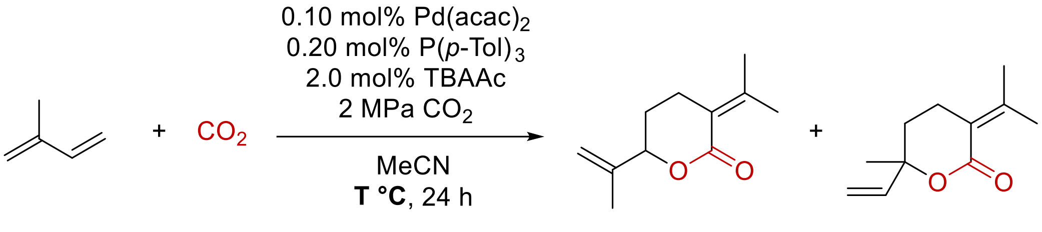


| Entry[a] | T (°C) | Yield **1a** + **1b**[b] | Ratio **1a** / **1b** | Selectivity (%)[c] | TON[d] |
|---|---|---|---|---|---|
| 1 | 70 | 17 | 1 : 2.0 | 78 | 82 |
| 2 | 80 | 8 | 1 : 3 | 60 | 41 |
| 3 | 90 | 6 | 1 : 3 | 85 | 31 |
| 4 | 60 | 5 | 1 : 4 | 69 | 26 |
| 5 | 50 | 10 | 1 : 2.0 | 68 | 48 |

[a] Reaction conditions: isoprene (25 mmol), $Pd(acac)_2$ (0.10 mol%), $P(p\text{-}Tol)_3$ (0.20 mol%), TBAAc (2.0 mol%), MeCN (2.5 mL), $CO_2$ (2 MPa), T °C, 24 h. [b] GC-FID yield with external standard *n*-decane. [c] Determined by GC-FID area ratio of **1** versus all isoprene-derived products. [d] n(**1a**+**1b**)/n(Pd).

**Table S8:** Screening of $CO_2$ pressure.

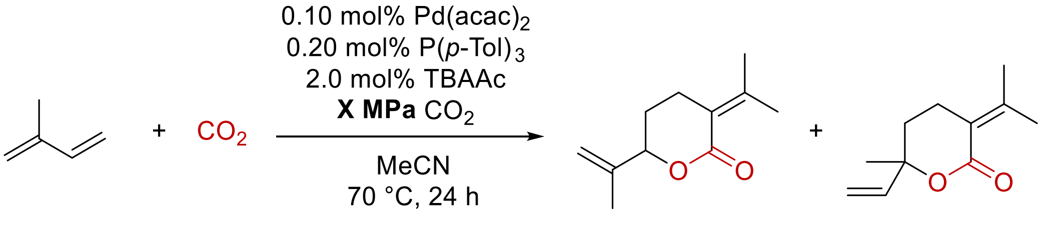


| Entry[a] | p($CO_2$) (MPa) | Yield **1a** + **1b**[b] | Ratio **1a** / **1b** | Selectivity (%)[c] | TON[d] |
|---|---|---|---|---|---|
| 1 | 2.0 | 17 | 1 : 2.0 | 78 | 82 |
| 2 | 4.0 | 19 | 1 : 2.0 | 81 | 96 |

[a] Reaction conditions: isoprene (25 mmol), $Pd(acac)_2$ (0.10 mol%), $P(p\text{-}Tol)_3$ (0.20 mol%), TBAAc (2.0 mol%), MeCN (2.5 mL), $CO_2$ (2 MPa), 70 °C, 24 h. [b] GC-FID yield with external standard *n*-decane. [c] Determined by GC-FID area ratio of **1** versus all isoprene-derived products. [d] n(**1a**+**1b**)/n(Pd).

### 1.5.2 Investigation of the influence of the water content in TBAAc on the reaction

Tetrabutylammonium acetate (TBAAc) is a hygroscopic compound that readily absorbs moisture from ambient air. In the initial optimization stage, we stored TBAAc in a desiccator and handled it under ambient conditions during weighting. Within a minute, the initially white solid visibly collects moisture to the point that it becomes a colorless liquid. By evacuating the autoclave after charging all solids, we attempted to remove all absorbed water from the reaction. This preparation method

afforded the desired products **1a** and **1b** in 17% yield (Table S9, entry 1).

In contrast, when anhydrous TBAAc obtained by rigorous drying was employed, no target products were formed. Instead, only isoprene-derived dimers and terpenes were detected by GC analysis (entries 2). Other commercial and self-synthesized batches produced the same results (entries 3-4). When anhydrous TBAAc was aged in the air, the reactivity was partially restored (entries 5-6), suggesting the importance of water in the reaction. When anhydrous TBAAc was exposed to air (25 °C, 60% rel. humidity) for 120 min, a colorless liquid was obtained. Water absorption is also evident by IR and NMR analysis, as shown in Fig. S6 below.

Defined amounts of water were then introduced as a stock solution in MeCN in the otherwise anhydrous conditions to determine the optimal amount of water for the reaction (entries 7-10). The optimal amount of water was found to be 0.2 mol% or twice the amount of palladium catalyst.

Note: The reaction is sensitive to the amount of water in the system and the ambient conditions. Over the course of six months we found that the optimal amount of water fluctuates between 0.2 and 0.5 mol%.

At this stage, the beneficial effect of TBAAc/water is unclear, but previous studies support two contrasting hypotheses. On the one hand, the addition of TBAAc and water promotes the reduction of Pd(II) to Pd(0), as shown by Wei et al.[4] On the other hand, the groups of Wang and Bao reported that $Pd(acac)_2$ is converted into Pd nanoparticles by the action of TBAAc. In their work, the water content in TBAAc is not discussed.[5,6]

**Table S9:** Screening of the water content in the reaction.

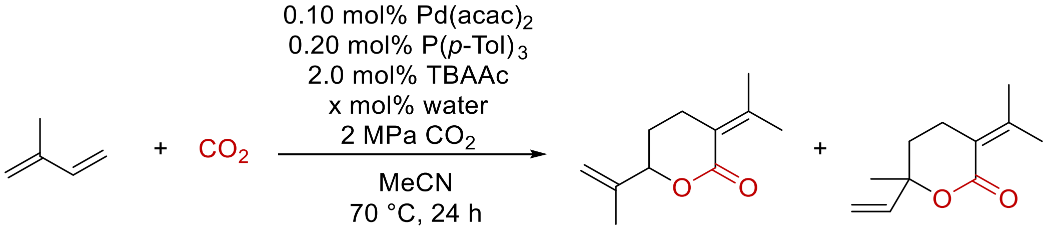


**1a** **1b**

| Entry[a] | TBAAc source | Water added | Yield **1a** + **1b**[b] | Ratio **1a** / **1b** | Selectivity (%)[c] | TON[d] |
|---|---|---|---|---|---|---|
| 1 | Commercial, wet | - | 17 | 1 : 2.0 | 78 | 82 |
| 2 | Commercial, vacuum-dried | - | 0 | n.d. | n.d. | n.d. |
| 3 | Commercial, anhydrous | - | 0 | n.d. | n.d. | n.d. |
| 4 | Self-synthesized, anhydrous | - | 5 | 1 : 1.8 | 46 | 27 |
| 5 | Commercial, anhydrous | Aged in the air for 15 min[e] | 9 | 1 : 3 | 59 | 45 |
| 6 | Commercial, anhydrous | Aged in the air for 90 min[e] | 3 | 1 : 7 | 50 | 16 |

| | | | | | | |
|---|---|---|---|---|---|---|
| 7 | Commercial, anhydrous | 0.10 mol%[f] | 0 | n.d. | n.d. | n.d. |
| 8 | Commercial, anhydrous | 0.20 mol%[f] | 19 | 1 : 1.8 | 77 | 93 |
| 9 | Commercial, anhydrous | 0.30 mol%[f] | 11 | 1 : 2.6 | 73 | 57 |
| 10 | Commercial, anhydrous | 0.50 mol%[f] | 3 | 1 : 2.1 | 18 | 12 |

[a] Reaction conditions: isoprene (25 mmol), $Pd(acac)_2$ (0.10 mol%), $P(p\text{-}Tol)_3$ (0.20 mol%), TBAAc (2.0 mol%), water (X mL, 0.25 M in MeCN), MeCN (2.3-X mL), $CO_2$ (2 MPa), 70 °C, 24 h. [b] GC-FID yield with external standard *n*-decane. [c] Determined by GC-FID area ratio of **1** versus all isoprene-derived products. [d] n(**1a**+**1b**)/n(Pd). [e] 25 °C, 60% rel. humidity. [f] Employed as 0.25 M stock solution of degassed deionized water in anhydrous acetonitrile.

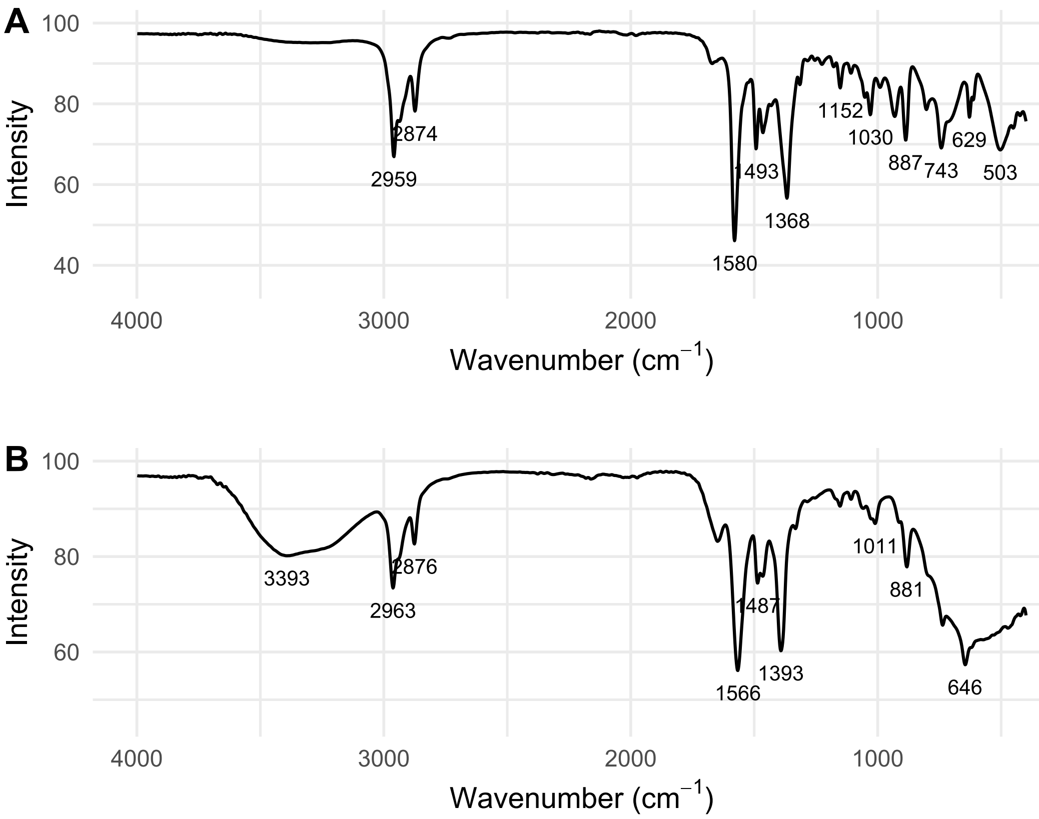


**Figure S6.** FTIR spectra of commercial TBAAc from Sigma Aldrich. A) stored in the desiccator. B) aged under air for 2 hours.

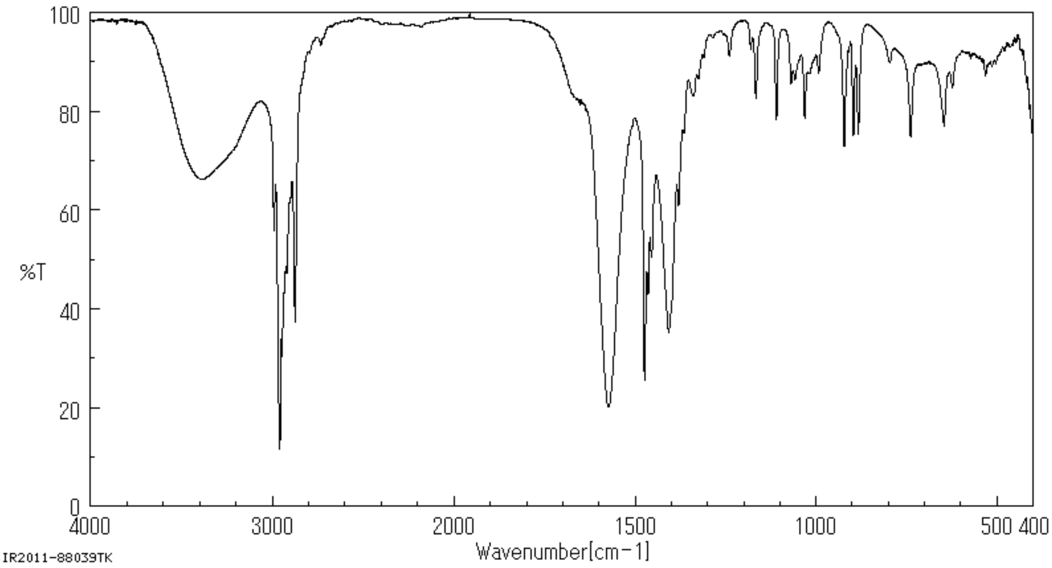


**Figure S7.** Literature FTIR spectrum of TBAAc [AIST SDBD].

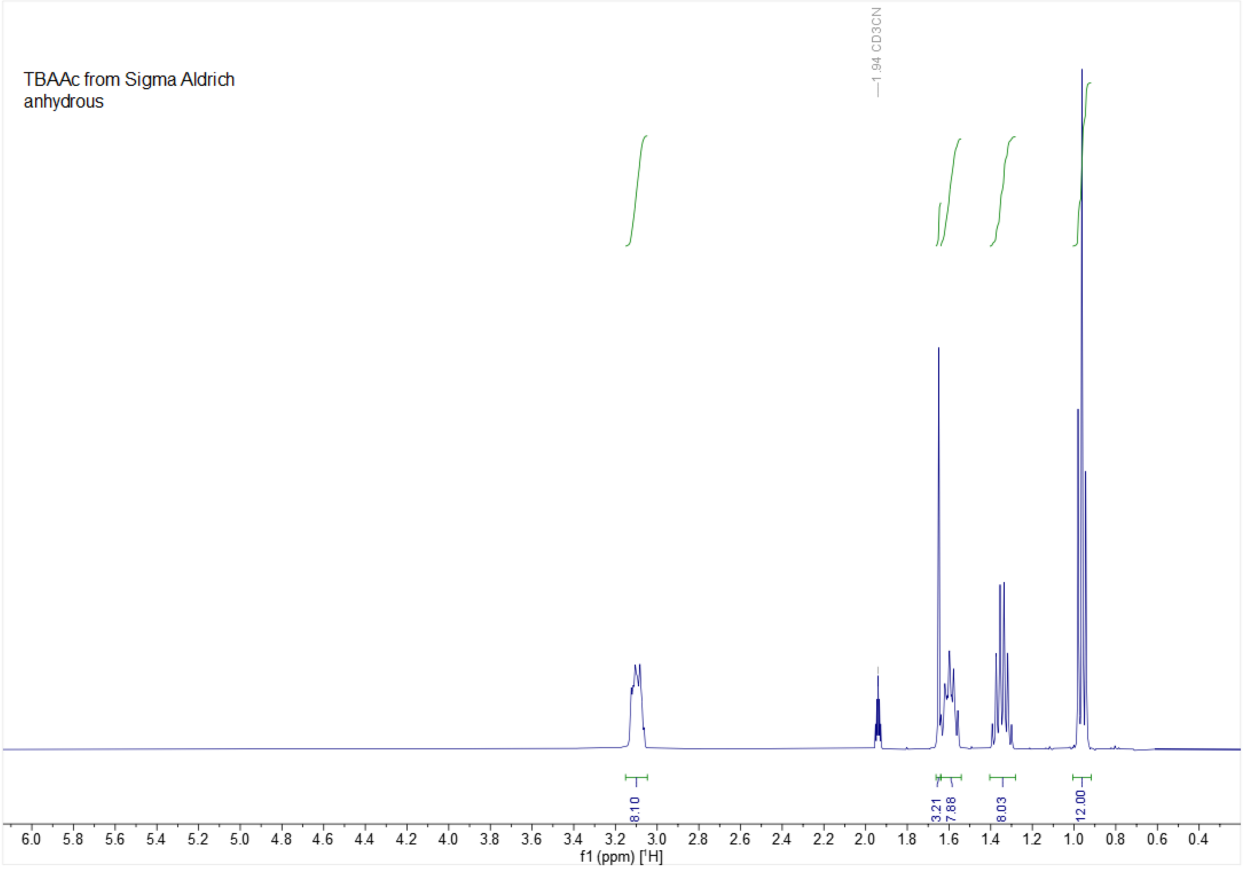


**Figure S8.** $^{1}$H NMR spectrum of commercial TBAAc from Sigma Aldrich stored in the desiccator in MeCN-$d_3$.

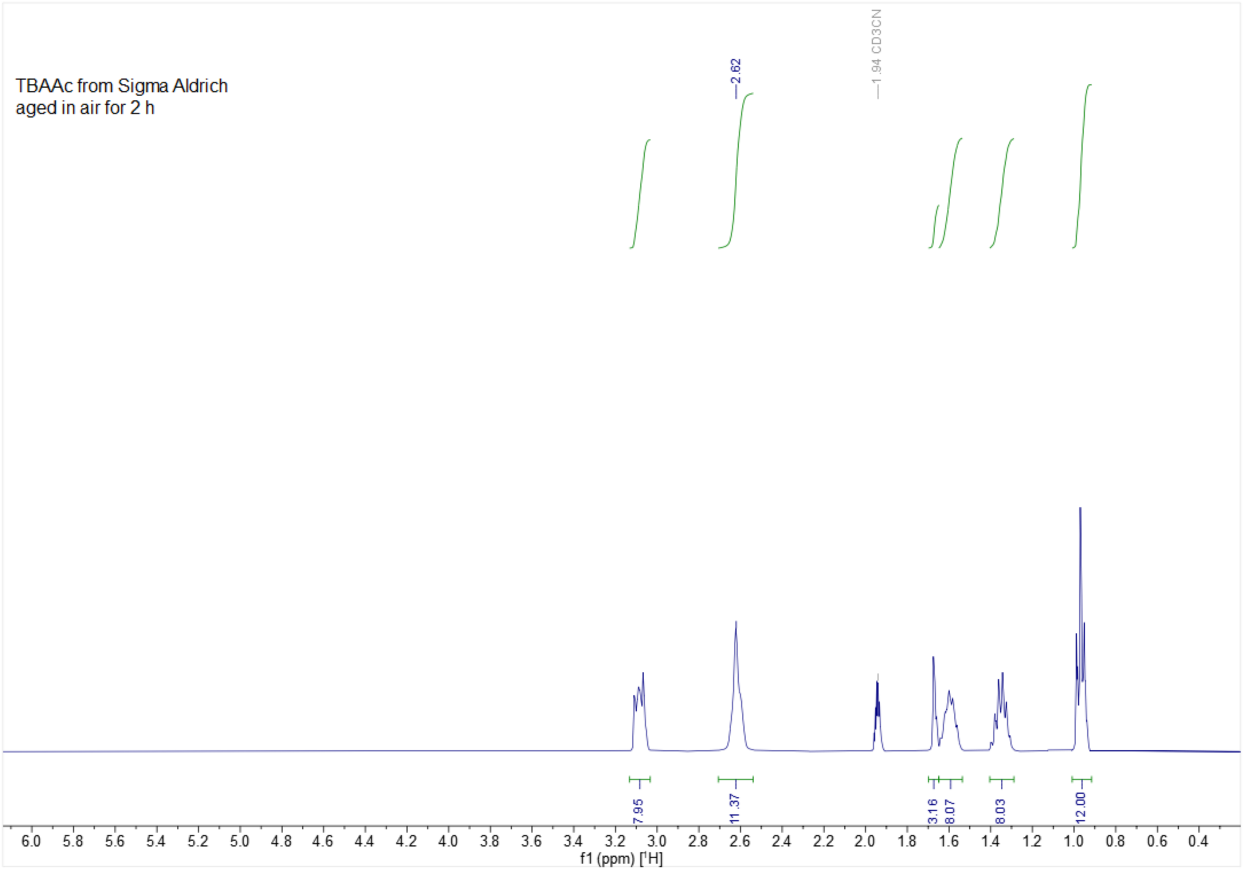


**Figure S9.** $^1$H NMR spectrum of commercial TBAAc from Sigma Aldrich aged in air for 2 hours in MeCN-$d_3$.

### 1.5.3 Optimization of Telomerization of isoprene and $CO_2$ with fixed water content

**Table S10:** Control experiments.

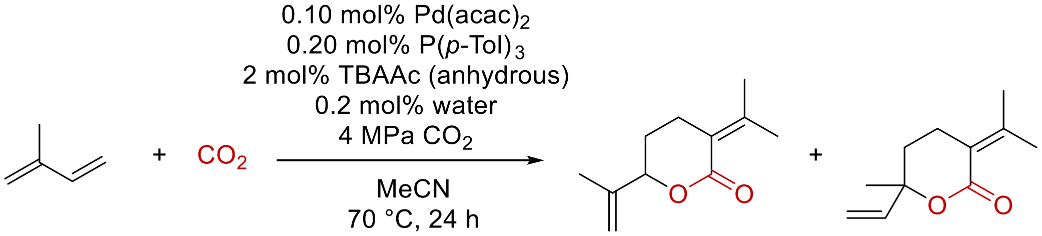


**1a** **1b**

| Entry[a] | Changes from above | Yield **1a** + **1b**[b] | Ratio **1a** / **1b** | Selectivity (%)[c] | TON[d] |
|---|---|---|---|---|---|
| 1 | None | 19 | 1 : 1.8 | 77 | 93 |
| 2 | Without Pd(acac)$_2$ | 0 | n.d. | n.d. | n.d. |
| 3 | without TBAAc | 0 | n.d. | n.d. | n.d. |
| 4 | without P(*p*-Tol)$_3$ | 2 | 1 : 12 | 46 | 11 |
| 5 | Without water | 0 | n.d. | n.d. | n.d. |
| 6 | $LaFe_{0.95}Pd_{0.05}O_3$ (LFPO)[7] instead of Pd(acac)$_2$ and P(*p*-Tol)$_3$ | <1 | 1 : 11 | n.d. | 1 |

| | | | | | |
|---|---|---|---|---|---|
| 7 | 0.3 mol% P(*p*-Tol)$_3$ | 6 | 1 : 4 | 63 | 31 |
| 8 | P(*p*-OMe-Ph)$_3$ instead of P(*p*-Tol)$_3$ | 8 | 1 : 4 | 68 | 38 |
| 9 | B$_2$pin$_2$ (1.0 mol%) instead of TBAAc and water | n.d. | n.d. | n.d. | n.d. |
| 10 | B$_2$pin$_2$ (0.1 mol%) instead of TBAAc and water | n.d. | n.d. | n.d. | n.d. |
| 11 | TBAOTf instead of TBAAc, without water | 0.2 | 1 : 1.8 | n.d. | 1 |

[a] Reaction conditions: isoprene (25 mmol), Pd(acac)$_2$ (0.10 mol%), P(*p*-Tol)$_3$ (0.20 mol%), TBAAc (2.0 mol%), water (0.2 mL, 0.20 mol%, 0.25 M in MeCN), MeCN (2.3 mL), $CO_2$ (4 MPa), 70 °C, 24 h. [b] GC-FID yield with external standard *n*-decane. [c] Determined by GC-FID area ratio of **1** versus all isoprene-derived products. [d] n(**1a**+**1b**)/n(Pd).

**Table S11:** Screening of extended reaction times at lower temperatures.

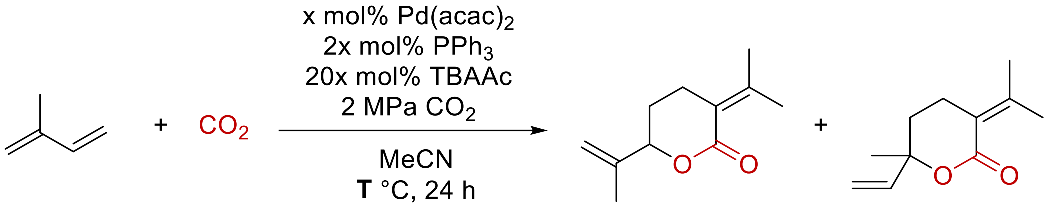


**1a** **1b**

| Entry[a] | T (°C) | Time (days) | Yield **1a** + **1b**[b] | Ratio **1a** / **1b** | Selectivity (%)[c] | TON[d] |
|---|---|---|---|---|---|---|
| 1 | 70 | 1 | 19 | 1 : 1.8 | 77 | 93 |
| 2 | 60 | 2 | 10 | 1 : 3.2 | 81 | 52 |
| 3 | 60 | 3 | 17 | 1 : 2.2 | 80 | 85 |
| 4 | 60 | 4 | 23 | 1 : 2.0 | 80 | 114 |
| 5 | 50 | 4 | 20 | 1 : 2.6 | 79 | 99 |

[a] Reaction conditions: isoprene (25 mmol), Pd(acac)$_2$ (0.10 mol%), P(*p*-Tol)$_3$ (0.20 mol%), TBAAc (2.0 mol%), water (0.2 mL, 0.20 mol%, 0.25 M in MeCN), MeCN (2.3 mL), $CO_2$ (4 MPa), 70 °C, 24 h. [b] GC-FID yield with external standard *n*-decane. [c] Determined by GC-FID area ratio of **1** versus all isoprene-derived products. [d] n(**1a**+**1b**)/n(Pd).

**Table S12:** Measurement of the conversion of isoprene as function of reaction time.

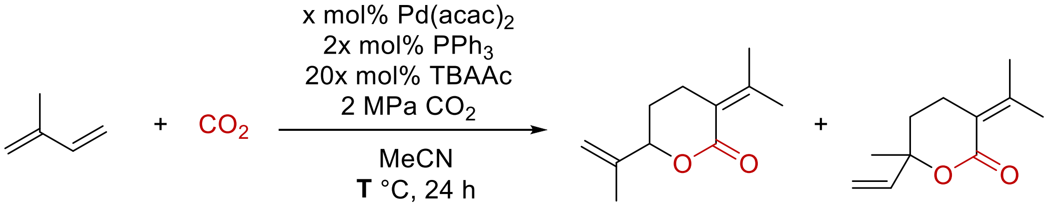


**1a** **1b**

| Entry[a] | Time (hours) | Yield **1a** + **1b**[b] | Conversion isoprene (%) |
|---|---|---|---|
| 1 | 8 | 4 | 55 |
| 2 | 24 | 8 | 59 |
| 3 | 48 | 22 | 60 |

[a] Reaction conditions: isoprene (25 mmol), $Pd(acac)_2$ (0.10 mol%), $P(p\text{-Tol})_3$ (0.20 mol%), TBAAc (2.0 mol%), water (0.5 mL, 0.50 mol%, 0.25 M in MeCN), MeCN (2.0 mL), $CO_2$ (2 MPa), 70 °C. [b] GC-FID yield with external standard *n*-decane. [c] Determined by GC-FID area ratio of **1** versus all isoprene-derived products. [d] n(**1a**+**1b**)/n(Pd).

## 1.6 Optimization of Polymerization of Lactone 1

**General Procedure for Neat Radical Polymerization:**

A mixture of lactone **1** (0.100 mmol) and initiator (0.0010 mmol) was stirred at 120 ºC for 24 hours in a 5 mL vial under argon atmosphere. Upon cooling, an aliquot was analyzed by $^1$H NMR spectroscopy to determine the conversion of **1**. The resulting mixture was diluted with an excess amount of methanol. No solids were obtained. Therefore, the solution was concentrated and analyzed by $^1$H NMR spectroscopy, which indicated a recovery of **1**.

**General Procedure for Emulsion Polymerization:**

A mixture of lactone **1** (0.50 mmol), KPS (0.050 mmol), and 15 mM SDS solution (2.5 mL) in a 25 mL Schlenk tube was degassed by sparging with nitrogen and stirred at 100 ºC for 24 hours under nitrogen atmosphere. The resulting mixture was diluted with an excess amount of methanol. No solids were obtained. Therefore, the solution was concentrated and analyzed by $^1$H NMR spectroscopy, which indicated a recovery of **1**.

**General Procedure for Zinc Chloride/Ethylene Carbonate Conditions:**

A mixture of lactone **1** (0.50 mmol), $ZnCl_2$ (0.50 mmol), V-40 (0.005 mmol), and ethylene carbonate (2.0 mmol) was stirred at 120 ºC for 24 hours in a 5 mL vial under argon atmosphere. Upon cooling, an aliquot was analyzed by $^1$H NMR spectroscopy to determine the conversion of **1**. The resulting solid mixture was diluted THF (0.5 mL) and precipitated into an excess amount of methanol (15 mL). The formed precipitate was collected and washed with methanol (2x5 mL). The remaining solid was dissolved in THF (1 mL) and reprecipitated with methanol (10 mL) to afford an off-white solid.

**General Procedure for Copolymerization with Ethylene:**

A 50 mL stainless steel autoclave equipped with a glass tube was dried in an oven at 120 °C and then allowed to cool inside a glovebox. The inner glass tube was charged with AIBN (0.10 mmol), dimethyl carbonate (5.0 mL), and **1** (0.50 mmol) and then placed in the autoclave. The autoclave was then sealed, charged with ethylene (0.7 MPa), and stirred in an isothermal heating block at 100 °C for 23 h. After cooling the autoclaves to room temperature, the excess gas pressure was carefully vented in a well-ventilated fume hood. The resulting mixture was diluted with an excess amount of methanol. No solids were obtained. Therefore, the solution was concentrated and analyzed by $^1$H NMR spectroscopy, which indicated recovery of **1**.

**General Procedure for Copolymerization with Methyl Methacrylate:**

A mixture of lactone **1** (0.50 mmol), methyl methacrylate (0.50 mmol), and AIBN (0.10 mmol) was stirred at 100 ºC for 20

hours in a 5 mL vial under an argon atmosphere. Upon cooling, an aliquot was analyzed by $^1$H NMR spectroscopy to determine the conversion of **1**. The resulting mixture was diluted with an excess amount of methanol. No solids were obtained. Therefore, the solution was concentrated and analyzed by $^1$H NMR spectroscopy, which indicated recovery of **1**.

**General Procedure for Organocatalytic Ring-Opening Polymerization:**

A mixture of lactone **1** (0.100 mmol), 1,5,7-triazabicyclo[4.4.0]dec-5-ene (TBD) (0.005 mmol) and 3-phenylpropanol (0.001 mmol) was stirred at rt for 3 days and at 120 ºC for 24 hours in a 5 mL vial under argon atmosphere. Upon cooling, an aliquot was analyzed by $^1$H NMR spectroscopy to determine the conversion of **1**, which indicated recovery of **1**.

**Table S13.** Initial screening of polymerization conditions.

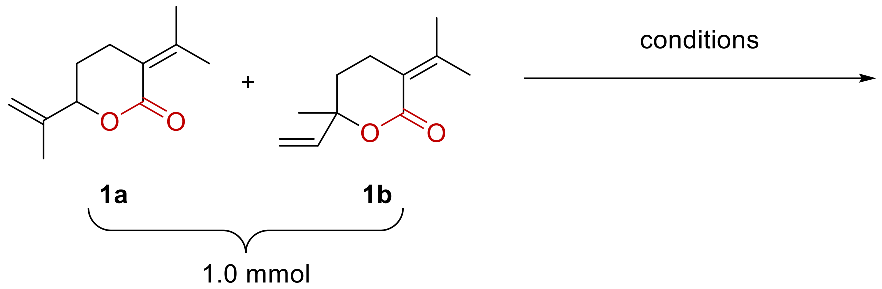


| Entry | Ref | Co-monomer | Polymerization method | Result |
|---|---|---|---|---|
| 1 | 8 | - | Neat radical polymerization under argon (180 °C, 4 days) | Monomer recovered |
| 2 | 8,9 | - | Neat radical polymerization under air (180 °C, 4 days) | Monomer recovered |
| 3 | 8 | - | Emulsion polymerization (100 °C, 4 days) | Monomer recovered |
| 4 | 8 | - | Zinc chloride/ethylene carbonate conditions (120 °C, 24 h) | Polymer |
| 5 | 10 | Ethylene | Neat radical polymerization (dimethyl carbonate, 100 °C, 23 h) | Monomer |
| 6 | 11 | Methyl methacrylate | Neat radical polymerization (100 °C, 20 h) | PMMA and monomer |
| 7 | 12 | - | Organocatalytic ring opening polymerization (TBD, $Ph(CH_2)_3OH$, 120 °C, 24 h) | Monomer |

### 1.6.1 Discussion of the structure of 2

The structural assignment of **2** was supported by both spectral data and comparison with the butadiene-derived polymer previously reported (dubbed poly-EVP).[8] When EVP was polymerized under neat conditions, one repeating unit with a bicyclic bridged motive (called α) was obtained. Meanwhile, when polymerization was carried out in the presence of $ZnCl_2$ and ethylene carbonate (i.e. similar to those used to synthesize **2**), three distinct repeating units (called α, β, γ) in the ratio 3:4:3 were identified. The possible repeating units of **2** were named in analogous fashion to aid comparison between the isoprene and the butadiene-derived polymers. In the same work, a co-polymer between butadiene, isoprene and $CO_2$ was also characterized. The NMR spectra of this material are highly complex, but the IR spectrum shows a major carbonyl peak at 1715 $cm^{-1}$ and minor ones at 1682 and 1645 $cm^{-1}$.

Two direct addition/propagation products can be envisioned by attack of a radical on the allylic olefin (left pathway) or the α,β-unsaturated ester (right pathway) (Fig. S10). Both intermediates can further react by two pathways to afford up to four possible repeating units. Intramolecular cyclization of the α-oxygen radical onto the conjugated olefin would result in bicyclic structure α. Meanwhile intermolecular radical addition would result in structure β. In case of the enolate-like radical, hydrogen atom transfer would lead to a stabilized allylic radical that could further react to structure γ. Otherwise, structure δ might be generated.

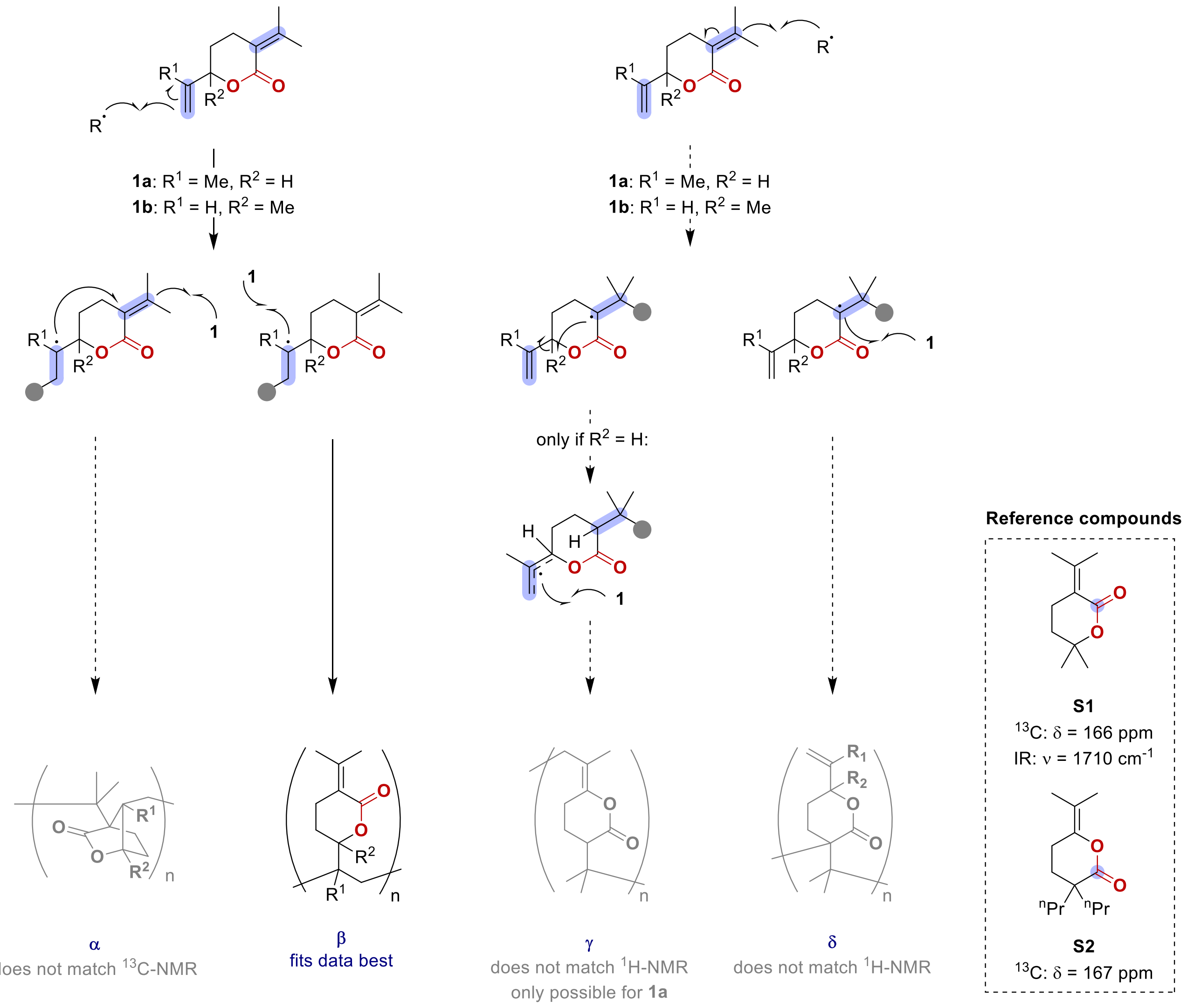


**Figure S10.** Assignment of the microstructure of **2**. Spectral data of **S1**[13] and **S2**.[14]

**Structure α**

The IR spectrum of **2** features one intense peak at 1719 $cm^{-1}$ that can be assigned to a cyclic conjugated ester. The signal is nearly unchanged from that of monomer **1** (1705 $cm^{-1}$), supporting that the moiety is retained in the polymer. In contrast, the carbonyl peak of saturated bicyclic structure α obtained in the case of EVP lies at 1769 $cm^{-1}$. The $^{13}C$ spectrum, specifically the carbonyl resonance at 169 ppm and the presence of olefinic signals at 135-145 ppm and 125 ppm rule out structure α whose carbonyl resonance resonates around 180 ppm.[8]

**Structure β**

Inspecting the IR spectrum of poly-EVP that contains motives α, β, γ, three carbonyl resonances were identified at 1761, 1713, and 1645 $cm^{-1}$, respectively. It is reasonable to assume that the highest wavenumber represents structure α. The signal at 1713 $cm^{-1}$, of poly-EVP corresponds to the one in **2**, which might be attributed to structures β or γ. This is also in agreement with reference compounds **S1** and **S2**.[13,14]

The shift of the carbonyl signal at 169 ppm could arise from a conjugated ester unit, as comparison with compound **S1** shows.[13] The absence of olefinic protons above 5 ppm with concurrent $sp^2$-hybridized signals in the $^{13}C$ spectrum support the assignment of a tetrasubstituted olefin, which is retained when the allylic olefin engages in polymerization. The signal at 4.3 ppm in the $^1H$ NMR spectrum can be assigned to the α-alkoxy proton in structure β arising from polymerization of the allylic ester in **1a**. In case of **1b**, all signals can be assigned in the aliphatic region. The IR spectrum does not allow to differentiate between **1a** and **1b**, which is assumed to be the case for **2** as well.

Apart from one broad signal at 3.7 ppm with integration of $^1H$ all signals can be assigned to structure β. While it is unclear which structure gives rise to this signal, an analogous signal can be found in the co-polymer of butadiene, isoprene and $CO_2$ (ref.8, SI, Figure 27).[8] Moreover, a signal at 3.5 ppm can also be found in the α,β,γ-type poly-EVP. It can be assumed that is an inherent byproduct of this lactone monomer class.

**Structure γ**

Attack on the tetrasubstituted conjugated olefin, followed by 1,5-HAT would lead to structure γ. While in agreement with the carbonyl shift of reference compounds **S2**,[14] this structure can also be excluded, as the olefin is sterically and electronically deactivated.[8,15] Further, radical transfer is only possible for isomer **1a**. In that case, the α-carbonyl proton is expected to resonate around 2 ppm, leaving the signal at 4.3 ppm unassigned.

**Structure δ**

This structure would arise from direct propagation of the radical intermediate leading to δ. The expected 1,1'-disubstituted olefin signal is missing in the $^1H$ NMR spectrum, also ruling out this structure.

# 2. Supplementary Discussion

## 2.1 Computational Details

The computations were performed using a workstation at the Research Center for Computational Science, National Institutes of Natural Sciences, Okazaki, Japan. Conformer searches for ground state structures were conducted with CREST (version 3.0.2) using default settings.[16] Additional conformers were added manually if necessary. Conformer searches for transition state structures were conducted manually based on the conformer ensembles of the corresponding ground state structures.

All DFT calculations were conducted using the Gaussian16 suite of programs (Revision C.02).[17] The keyword integral(grid=ultrafine) was used in all calculations to limit grid-based errors.[18] Optimizations were conducted at the PBE0 level of theory,[19–21] including Grimme's dispersion correction (D3) with Becke-Johnson damping[22,23] and the SMD solvent model for acetonitrile.[24] All atoms except Pd were modeled with the def2svp basis set and Pd was modeled with def2tzvp basis set. Frequency calculations were carried out with the optimized geometries using the same level of theory and thermal correction to 341.15 K. Ground states were identified by having zero imaginary frequencies in frequency calculations, whereas all reported transition states have exactly one imaginary frequency corresponding to the appropriate reactivity. For all transition states, Intrinsic Reaction Coordinate (IRC) were calculated to confirm that they indeed connected between correct minima. Quasi-harmonic vibrational corrections[25] were applied using the method proposed by Grimme[26] (cut-off value of 100 $cm^{-1}$) using the GoodVibes program.[27] Single point energies were calculated with the PBE0 functional, including Grimme dispersion correction (D3) with Becke-Johnson damping and the SMD solvent model for acetonitrile. The functionals M06-2X and ωB97X-D were also used for single-point energy calculations to evaluate the obtained energies. All atoms except Pd were modeled with the def2tzvp basis set and Pd was modeled with def2qzvp basis set. Structures were visualized with CYLview.[28] Energy diagrams were created using EveRplot (version 1.1).[29]

## 2.2 Discussion of the free energy diagram of the reaction

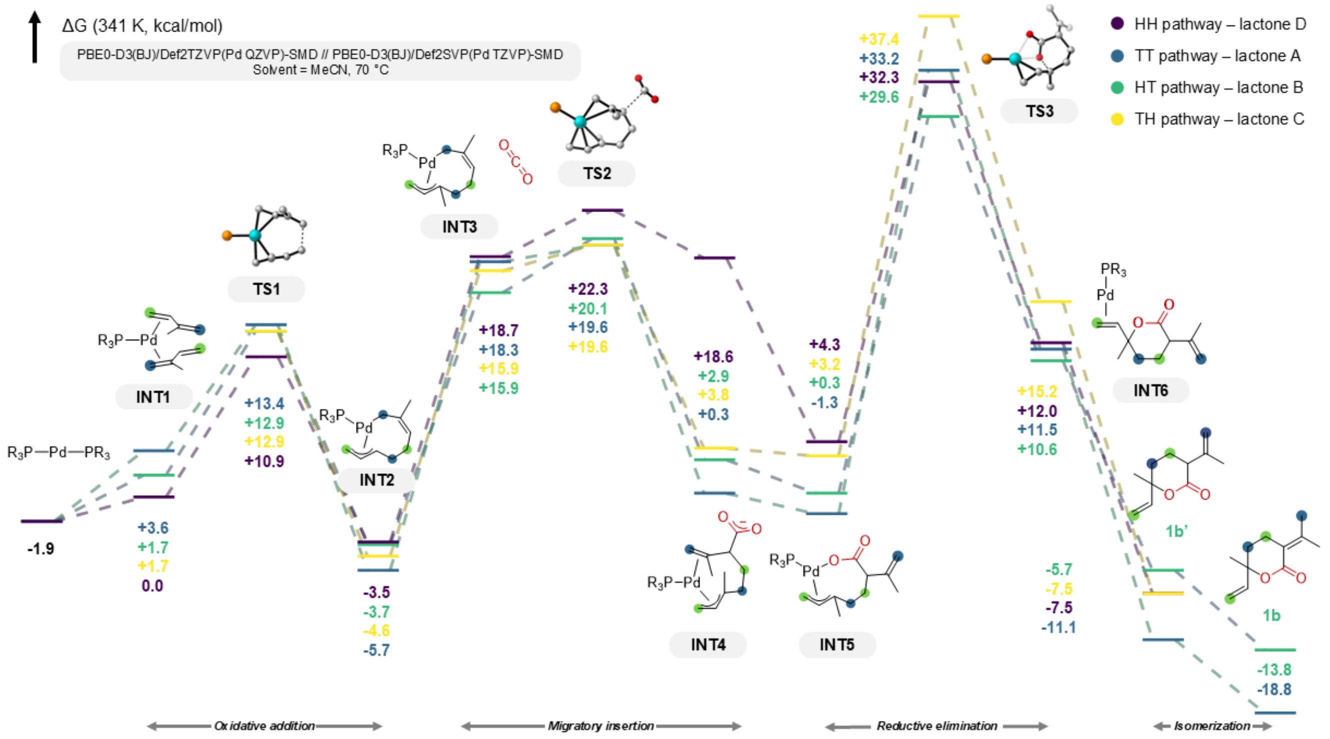


**Figure S11**. Calculated free energy diagram at the PBE0-D3(BJ)/Def2TZVP(Pd QZVP)-SMD // PBE0-D3(BJ)/Def2SVP(Pd TZVP)-SMD level of theory. Hydrogens and the aryl groups of the phosphine ligand are omitted for clarity.

## 2.3 Benchmarking

We calculated single point energies of several structures using a selection of functionals (Table S14) to see if the different methods give similar results. The energies are separated by a 5 kcal/mol window with the energies calculated with the ωB97X-D functional being the highest. The highest barrier (**TS3**) ranges between +26.5 kcal/mol for M06-2X and +34.6 kcal/mol for ωB97X-D.

**Table S14**. Calculated Gibbs free energies with quasi-harmonic correction using different functionals in the single point calculation relative to the energy of **INT1-HH**. The geometries were optimized at the PBE0-D3(BJ)/Def2SVP(Pd TZVP)-SMD level of theory. All values in kcal/mol.

| | PBE0-D3(BJ) | ωB97X-D | M06-2X |
|---|---|---|---|
| **INT1-HH** | 0 | 0 | 0 |
| **INT1-HT** | 1.7 | 1.7 | 1.2 |
| **TS1-HT** | 12.9 | 18.1 | 15.5 |
| **INT2-HT** | -3.7 | -1.8 | 0.3 |
| **INT3-HT** | 17.2 | 20.1 | 19.5 |
| **TS2-HT** | 20.1 | 26.9 | 26.0 |
| **INT4-HT** | 3.3 | 6.9 | 7.6 |
| **INT5-HT** | 0.3 | 2.7 | -1.9 |

| | | | |
|---|---|---|---|
| **TS3-HT** [a] | 29.6 | 34.6 | 26.5 |
| **INT6-HT** | 10.6 | 10.9 | 5.1 |

[a] The structure showed a small persistent imaginary frequency (<10 cm$^{-1}$).

## 2.4 Summary of Calculated Energy Values

**Table S15:** Summary of calculated energy values. $E_{SPC}$: electronic energy of the single point calculation. E: electronic energy of the optimization. ZPE: zero-point energy correction. $H_{SPC}$: enthalpy of the single point calculation. T.S: entropy term. T.qh-S: entropy term with quasi-harmonic energy correction. $G(T)_{SPC}$: free energy without quasi-harmonic correction, calculated as [$G(T)_{SPC}$] = [HSPC] – [T.S]. qh-$G(T)_{SPC}$: free energy with quasi-harmonic correction, calculated as [qh-$G(T)_{SPC}$] = [$H_{SPC}$] – [T.qh-S]. All values in Hartree.

| | $E_{SPC}$ | E | ZPE | $H_{SPC}$ | T.S | T.qh-S | $G(T)_{SPC}$ | qh-$G(T)_{SPC}$ |
|---|---|---|---|---|---|---|---|---|
| **Isoprene** | -195.14436 | -194.93549 | 0.11329 | -195.02252 | 0.04245 | 0.04244 | -195.06497 | -195.06496 |
| **$CO_2$** | -188.46735 | -188.24446 | 0.01189 | -188.45123 | 0.02864 | 0.02864 | -188.47987 | -188.47987 |
| **P(*p*Tol)$_3$** | -1153.51002 | -1152.54872 | 0.35673 | -1153.12543 | 0.09502 | 0.08639 | -1153.22045 | -1153.21182 |
| **1a** | -578.81477 | -578.81477 | 0.24877 | -578.54715 | 0.06831 | 0.06579 | -578.61546 | -578.61294 |
| **1b** | -578.81304 | -578.18809 | 0.24837 | -578.54573 | 0.06771 | 0.06566 | -578.61344 | -578.61139 |
| **1a'** | -578.80602 | -578.17918 | 0.24944 | -578.53784 | 0.06892 | 0.06587 | -578.60676 | -578.60372 |
| **1b'** | -578.80466 | -578.17811 | 0.24921 | -578.53696 | 0.06680 | 0.06464 | -578.60377 | -578.60160 |
| **1c** | -578.80089 | -578.17488 | 0.24954 | -578.53290 | 0.06699 | 0.06452 | -578.59989 | -578.59742 |
| **1d** | -578.80091 | -578.17491 | 0.24876 | -578.53358 | 0.06640 | 0.06445 | -578.59998 | -578.59804 |
| **$L_2$Pd** | -2435.02838 | -2433.10226 | 0.71571 | -2434.25136 | 0.17830 | 0.15448 | -2434.42965 | -2434.40584 |
| **INT1-HH** | -1671.83031 | -1670.45255 | 0.58787 | -1671.19370 | 0.14101 | 0.12719 | -1671.33471 | -1671.32090 |
| **INT1-HT** | -1671.82821 | -1670.45088 | 0.58799 | -1671.19158 | 0.13954 | 0.12655 | -1671.33113 | -1671.31814 |
| **INT1-TT** | -1671.82509 | -1670.44807 | 0.58792 | -1671.18847 | 0.13940 | 0.12669 | -1671.32787 | -1671.31515 |
| **TS1-HH** | -1671.81478 | -1670.43882 | 0.58813 | -1671.17923 | 0.13649 | 0.12430 | -1671.31572 | -1671.30354 |
| **TS1-HT** | -1671.81155 | -1670.43557 | 0.58830 | -1671.17587 | 0.13729 | 0.12452 | -1671.31316 | -1671.30040 |
| **TS1-TT** | -1671.81003 | -1670.43392 | 0.58798 | -1671.17460 | 0.13826 | 0.12502 | -1671.31286 | -1671.29962 |
| **INT2-HH** | -1671.84258 | -1670.46967 | 0.59192 | -1671.20388 | 0.13456 | 0.12266 | -1671.33844 | -1671.32655 |
| **INT2-HT** | -1671.84354 | -1670.47050 | 0.59207 | -1671.20478 | 0.13325 | 0.12209 | -1671.33803 | -1671.32687 |
| **INT2-TH** | -1671.84395 | -1670.47080 | 0.59171 | -1671.20538 | 0.13533 | 0.12286 | -1671.34071 | -1671.32824 |
| **INT2-TT** | -1671.84567 | -1670.47246 | 0.59183 | -1671.20702 | 0.13570 | 0.12301 | -1671.34272 | -1671.33002 |
| **INT3-HH** | -1860.29011 | -1858.69575 | 0.60282 | -1859.63458 | 0.15317 | 0.13633 | -1859.78775 | -1859.77091 |
| **INT3-HT** | -1860.29521 | -1858.70040 | 0.60326 | -1859.63951 | 0.15224 | 0.13593 | -1859.79175 | -1859.77544 |
| **INT3-TH** | -1860.29521 | -1858.70040 | 0.60326 | -1859.63952 | 0.15232 | 0.13597 | -1859.79184 | -1859.77548 |
| **INT3-TT** | -1860.29185 | -1858.69693 | 0.60312 | -1859.63635 | 0.15084 | 0.13518 | -1859.78719 | -1859.77153 |
| **TS2-HH** | -1860.28751 | -1858.69303 | 0.60344 | -1859.63297 | 0.14710 | 0.13227 | -1859.78006 | -1859.76524 |
| **TS2-HT** | -1860.29052 | -1858.69557 | 0.60302 | -1859.63613 | 0.14888 | 0.13337 | -1859.78501 | -1859.76950 |
| **TS2-TH** | -1860.29112 | -1858.69657 | 0.60291 | -1859.63691 | 0.14727 | 0.13263 | -1859.78418 | -1859.76953 |
| **TS2-TT** | -1860.29054 | -1858.69566 | 0.60274 | -1859.63639 | 0.14832 | 0.13318 | -1859.78471 | -1859.76957 |
| **INT4-HH** | -1860.29009 | -1858.69574 | 0.60277 | -1859.63459 | 0.15351 | 0.13649 | -1859.78809 | -1859.77107 |
| **INT4-HT** | -1860.32511 | -1858.72537 | 0.60830 | -1859.66668 | 0.14234 | 0.12954 | -1859.80902 | -1859.79621 |
| **INT4-TH** | -1860.32333 | -1858.72285 | 0.60785 | -1859.66540 | 0.14245 | 0.12928 | -1859.80786 | -1859.79468 |
| **INT4-TT** | -1860.32794 | -1858.72783 | 0.60777 | -1859.66979 | 0.14436 | 0.13052 | -1859.81415 | -1859.80031 |
| **INT5-HH** | -1860.32132 | -1858.73239 | 0.60760 | -1859.66339 | 0.14512 | 0.13051 | -1859.80850 | -1859.79390 |

| | | | | | | | | |
|---|---|---|---|---|---|---|---|---|
| **INT5-HT** | -1860.32791 | -1858.73792 | 0.60775 | -1859.66976 | 0.14495 | 0.13059 | -1859.81471 | -1859.80035 |
| **INT5-TH** | -1860.32232 | -1858.73383 | 0.60698 | -1859.66473 | 0.14529 | 0.13097 | -1859.81002 | -1859.79570 |
| **INT5-TT** | -1860.32889 | -1858.73940 | 0.60707 | -1859.67116 | 0.14632 | 0.13160 | -1859.81748 | -1859.80277 |
| **TS3-HH** [a] | -1860.27663 | -1858.69005 | 0.60577 | -1859.62189 | 0.14116 | 0.12743 | -1859.76305 | -1859.74933 |
| **TS3-HT** [a] | -1860.28162 | -1858.69488 | 0.60627 | -1859.62654 | 0.14059 | 0.12707 | -1859.76714 | -1859.75361 |
| **TS3-TH** | -1860.26802 | -1858.64739 | 0.60755 | -1859.61043 | 0.14612 | 0.13068 | -1859.75655 | -1859.74111 |
| **TS3-TT** | -1860.27291 | -1858.68630 | 0.60616 | -1859.61655 | 0.14746 | 0.13124 | -1859.76401 | -1859.74779 |
| **INT6-HH** | -1860.31054 | -1858.72532 | 0.60882 | -1859.65154 | 0.14508 | 0.13011 | -1859.79662 | -1859.78165 |
| **INT6-HT** | -1860.31304 | -1858.727285 | 0.60841 | -1859.65458 | 0.14338 | 0.12937 | -1859.79796 | -1859.78394 |
| **INT6-TH** | -1860.30453 | -1858.71818 | 0.60842 | -1859.64574 | 0.14637 | 0.13075 | -1859.79210 | -1859.77648 |
| **INT6-TT** | -1860.31148 | -1858.72562 | 0.60855 | -1859.65273 | 0.14354 | 0.12976 | -1859.79627 | -1859.78249 |

[a]: The structure showed a small persistent imaginary frequency (<10 $cm^{-1}$).

# 3. Supplementary Notes

## 3.1 NMR spectra

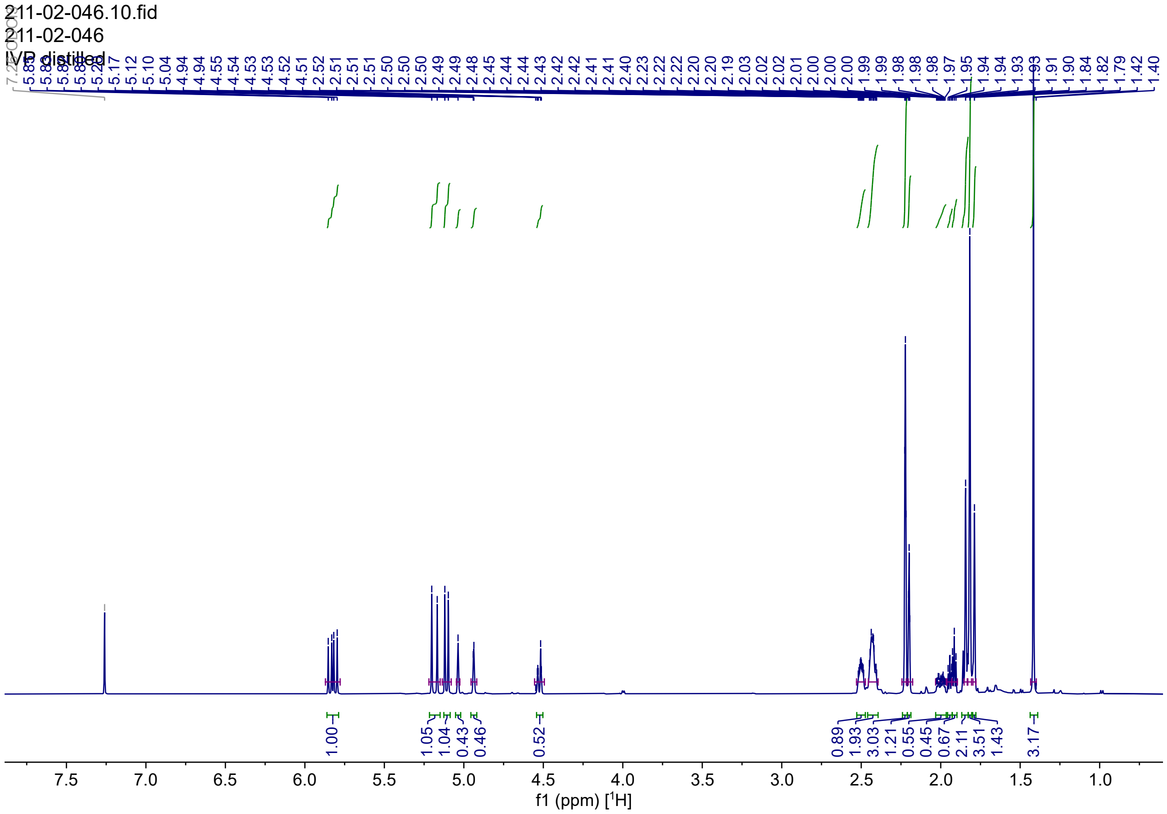

**Figure S12.** $^1$H NMR spectrum (500 MHz, $CDCl_3$) of lactone mixture **1a** and **1b**.

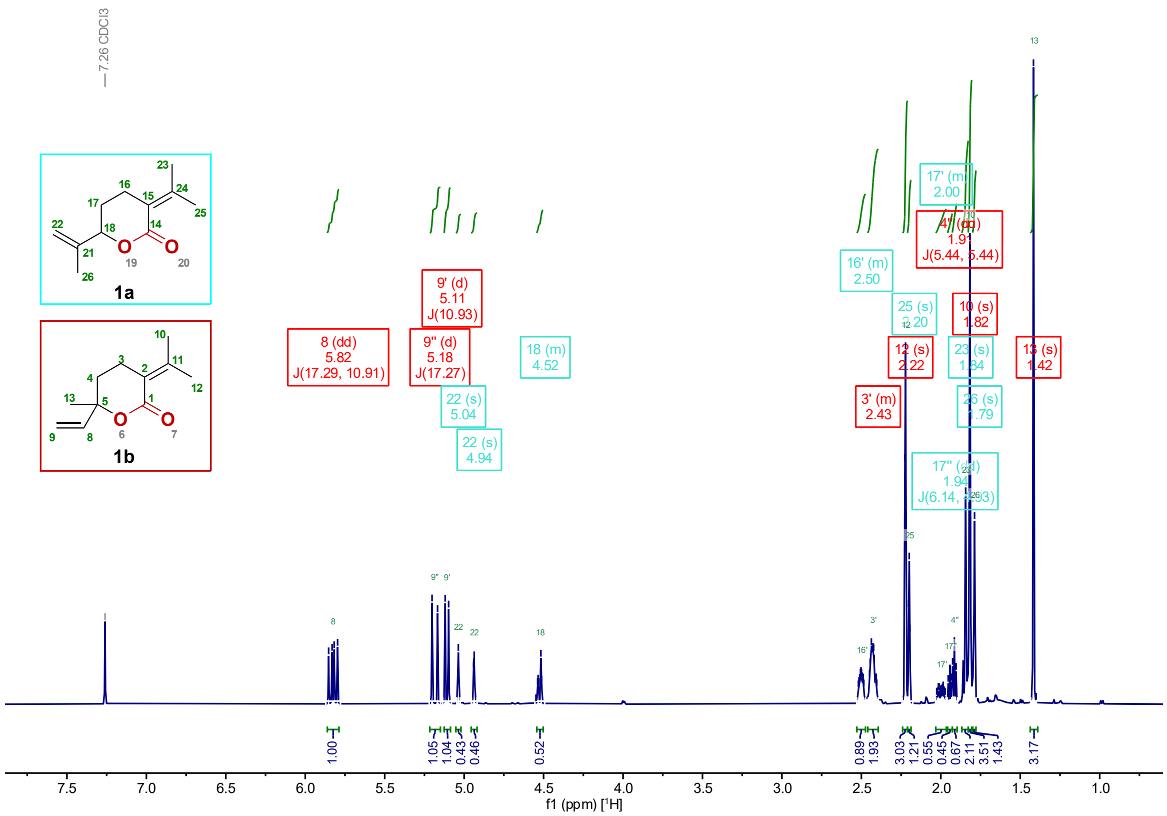

**Figure S13.** Assigned $^1$H NMR spectrum (500 MHz, $CDCl_3$) of lactone mixture **1a** and **1b**.

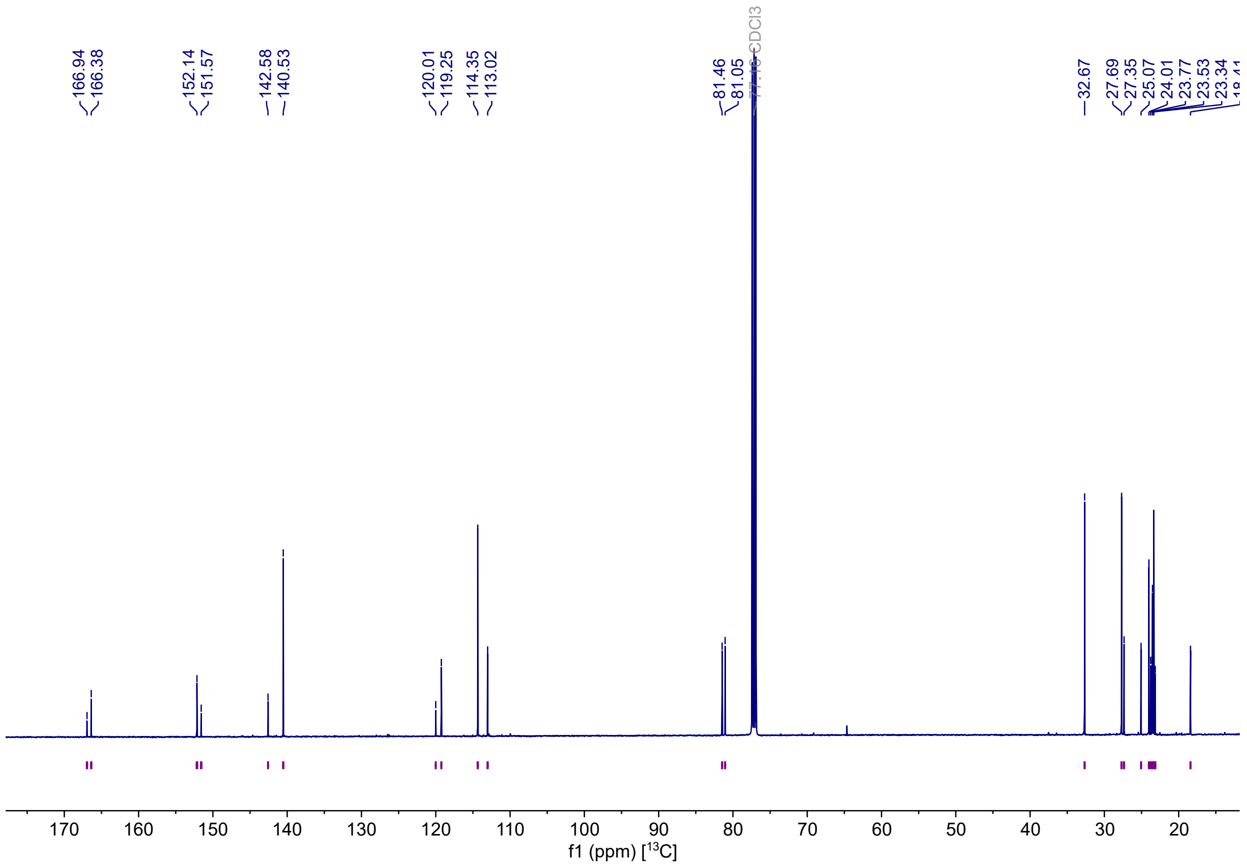


**Figure S14.** $^{13}C\{^1H\}$ NMR spectrum (126 MHz, $CDCl_3$) of lactone mixture **1a** and **1b**.

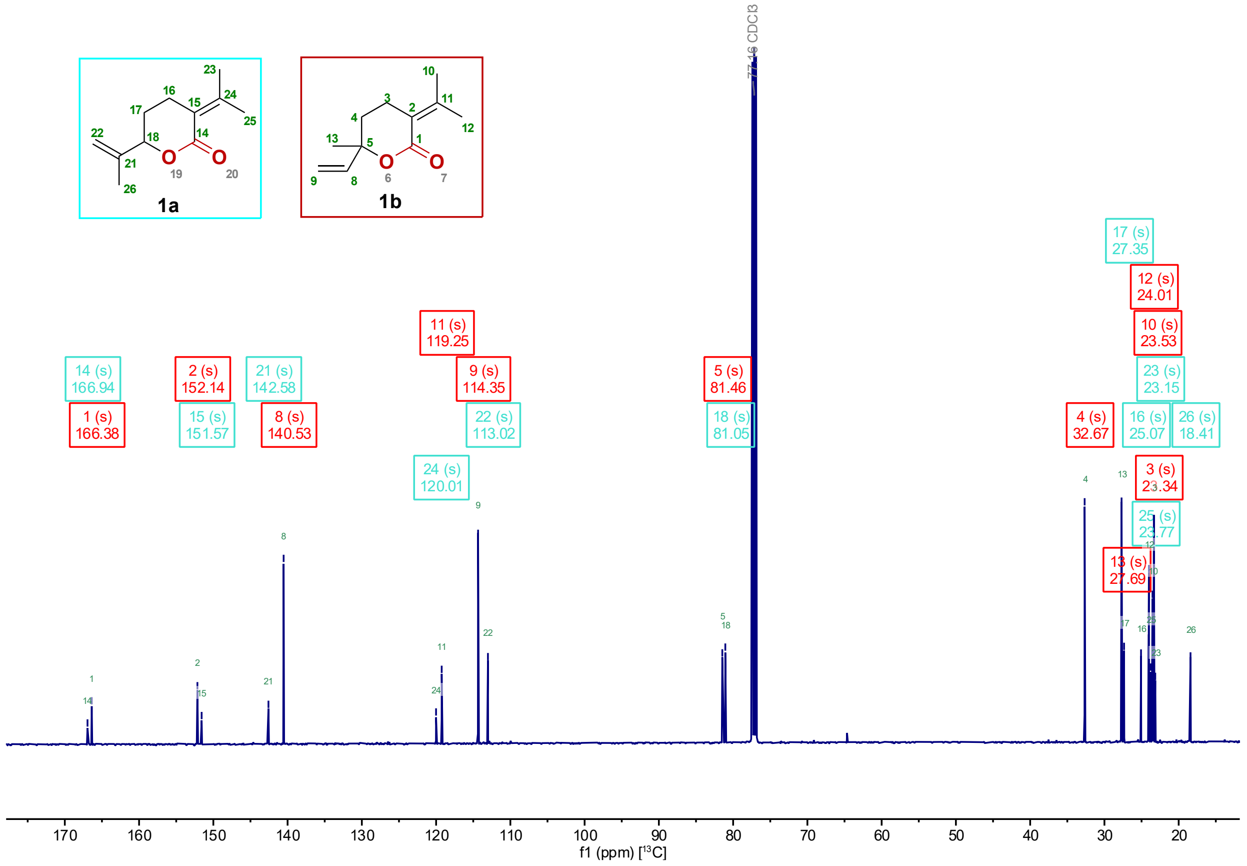


**Figure S15.** Assigned $^{13}C\{^1H\}$ NMR spectrum (126 MHz, $CDCl_3$) of lactone mixture **1a** and **1b**.

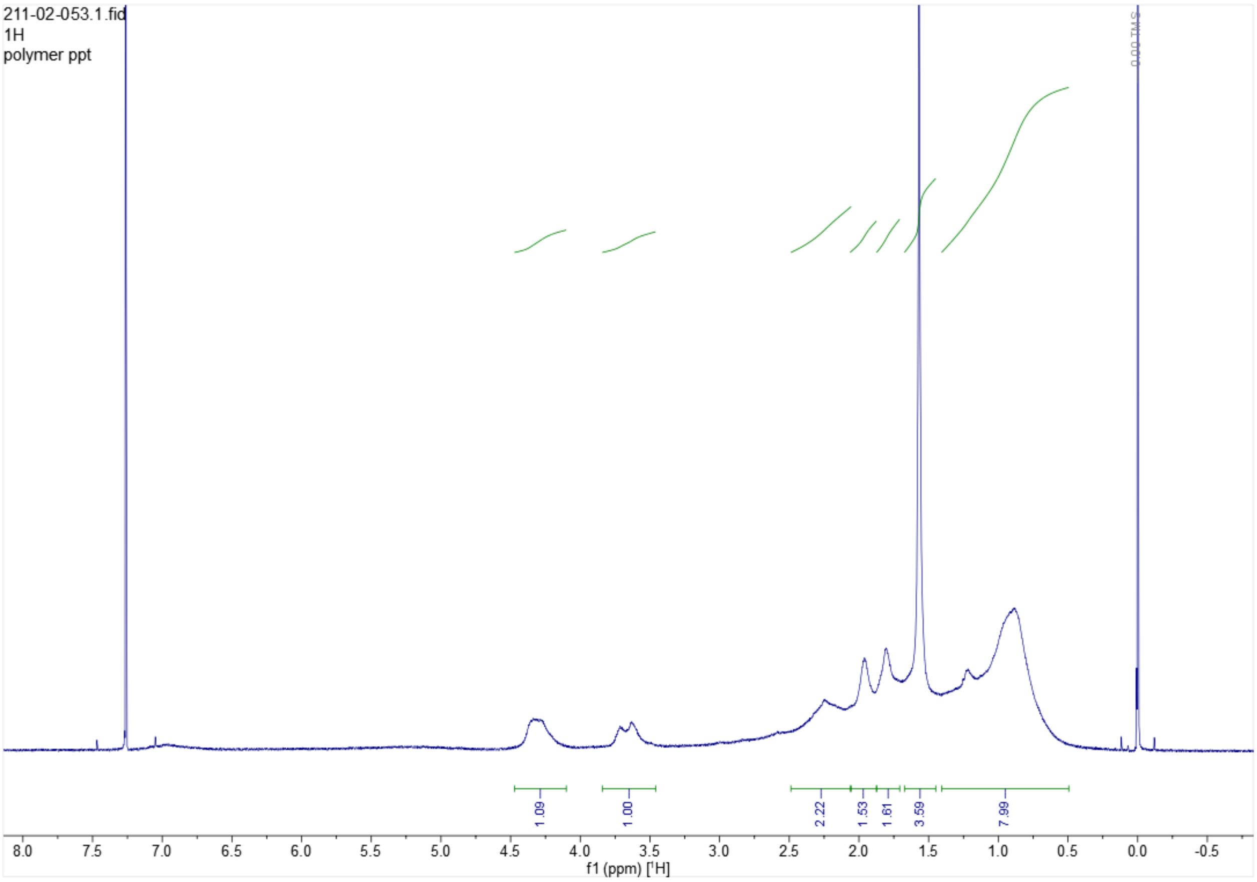


**Figure S16.** $^{1}H$ NMR spectrum (500 MHz, $CDCl_3$) of $CO_2$/isoprene copolymer **2**.

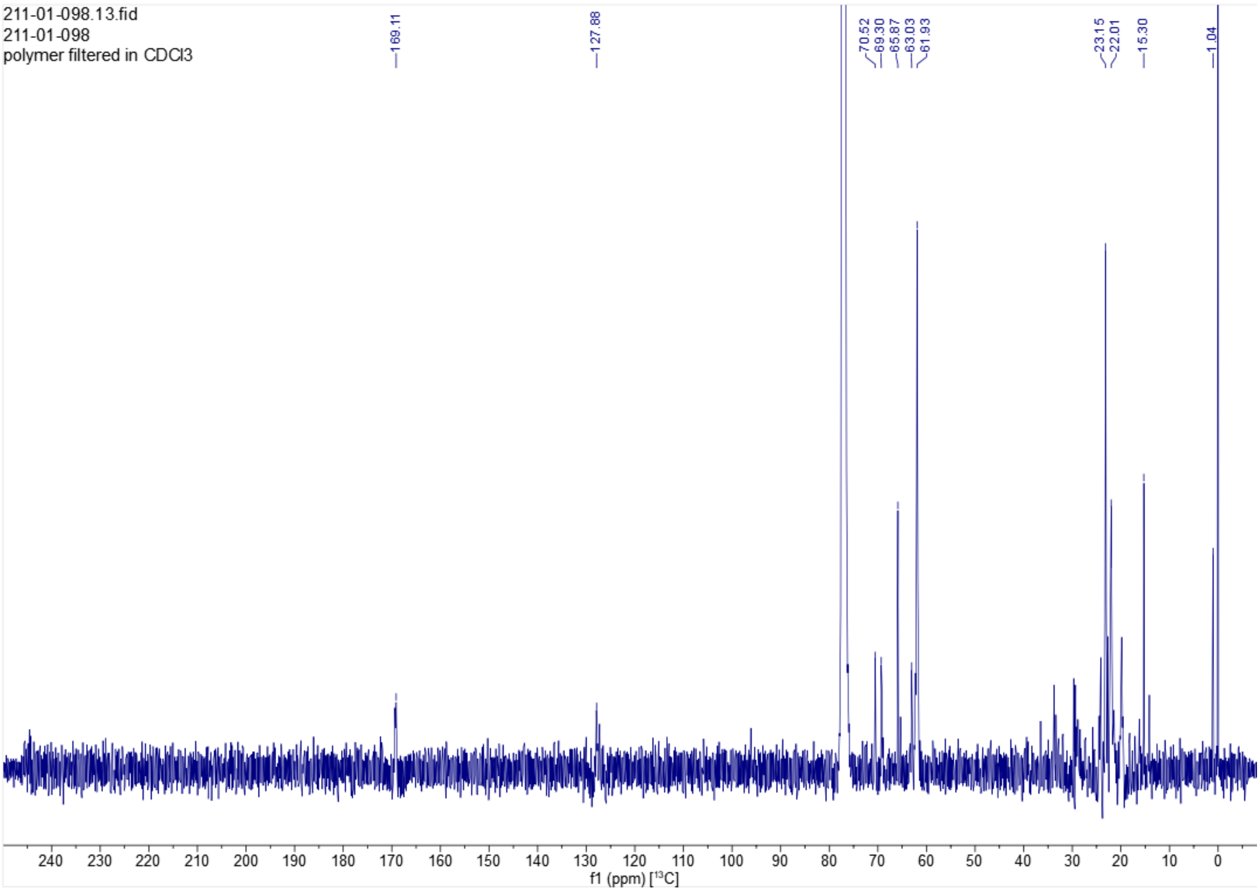


**Figure S17.** $^{13}C$ NMR spectrum (126 MHz, $CDCl_3$) of $CO_2$/isoprene copolymer **2**.

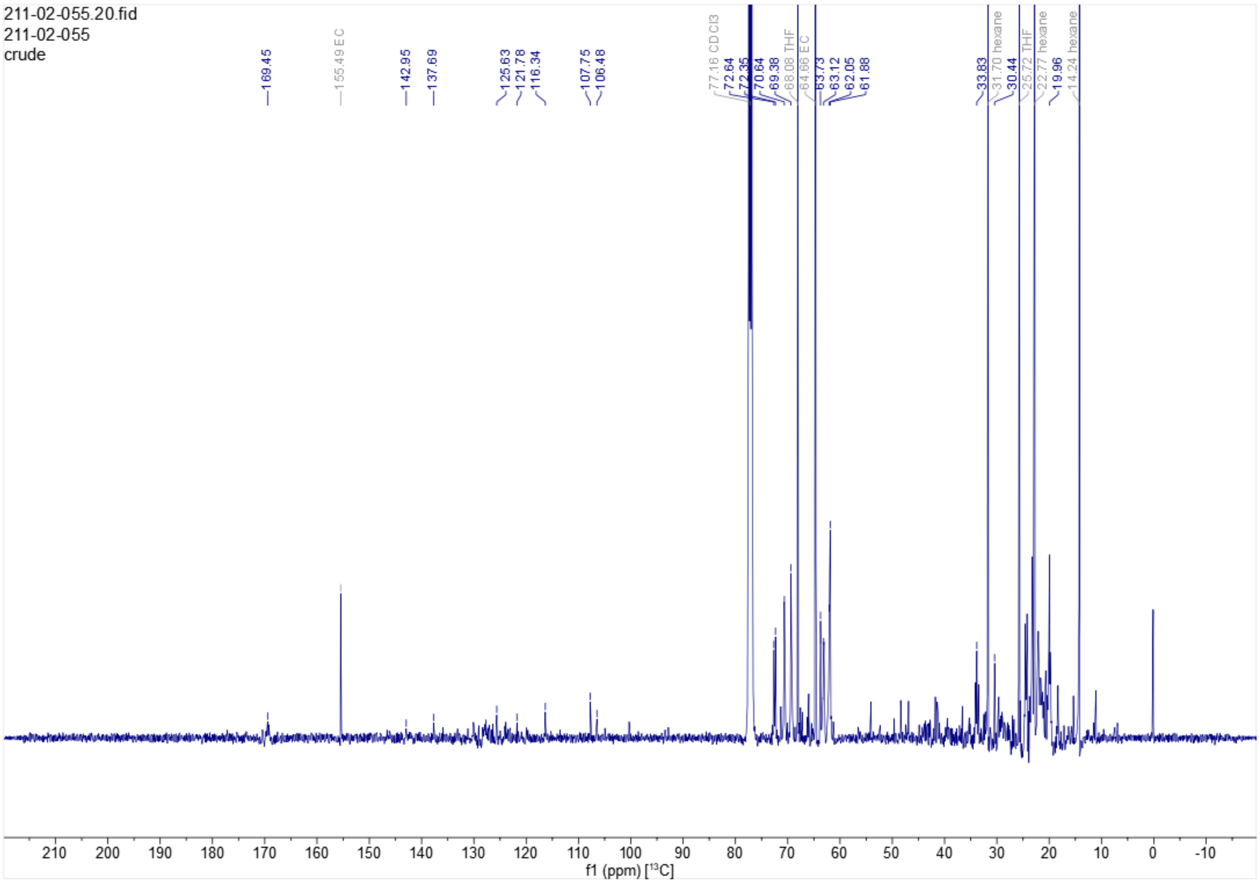


**Figure S18.** $^{13}$C NMR spectrum (126 MHz, $CDCl_3$) of $CO_2$/isoprene copolymer **2**. To increase sensitivity, samples containing residual solvent (THF, hexane) and ethylene carbonate (EC) were combined.

## 3.2 IR spectra

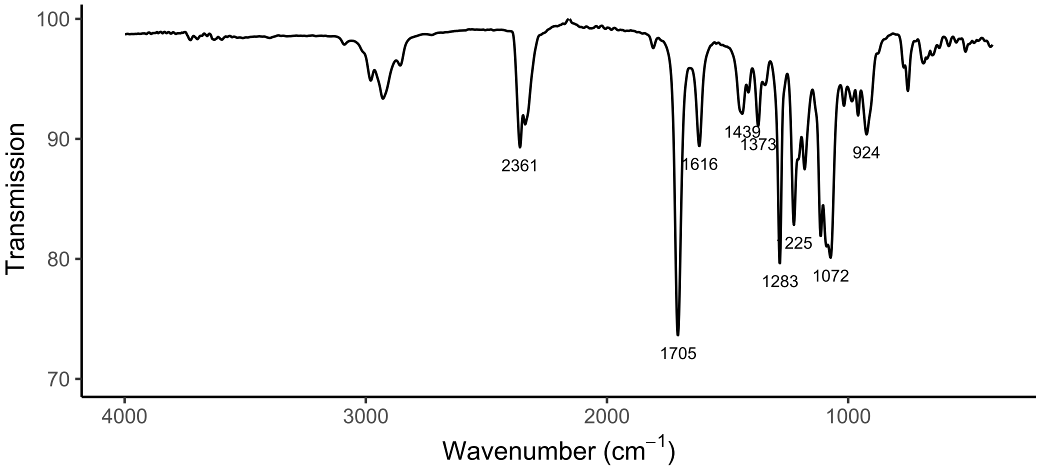


**Figure S19.** FTIR spectrum of monomer **1**.

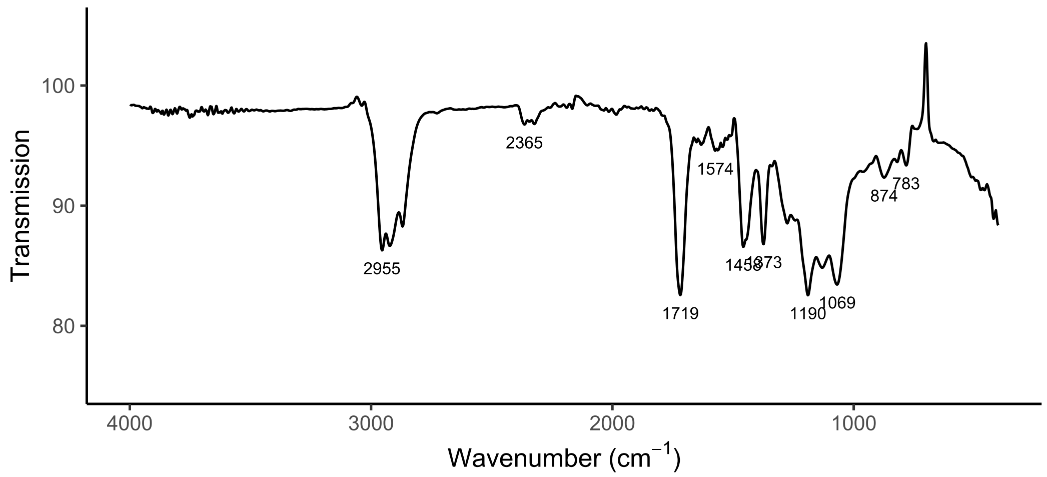


**Figure S20.** FTIR spectrum of $CO_2$/isoprene copolymer **2**.

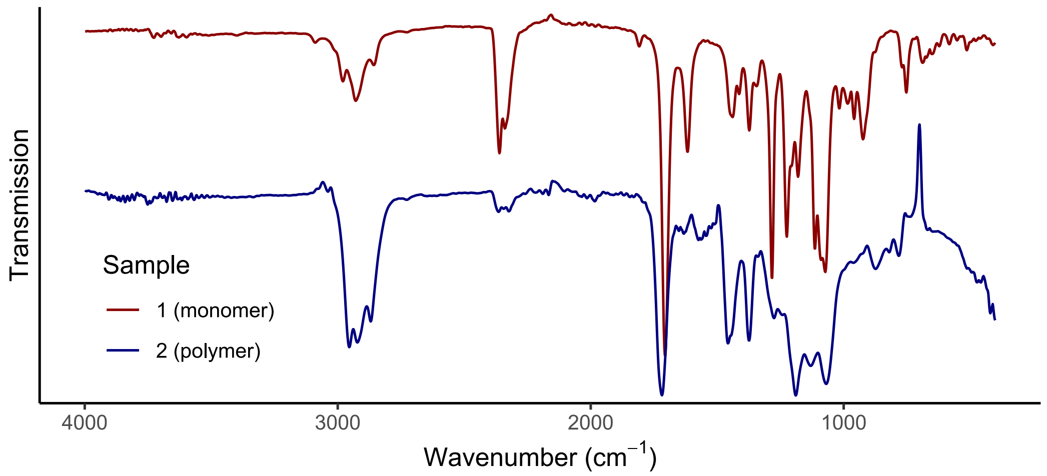


**Figure S21.** Stacked FTIR spectra of **1** and **2**.

## 3.3 GC-MS spectra

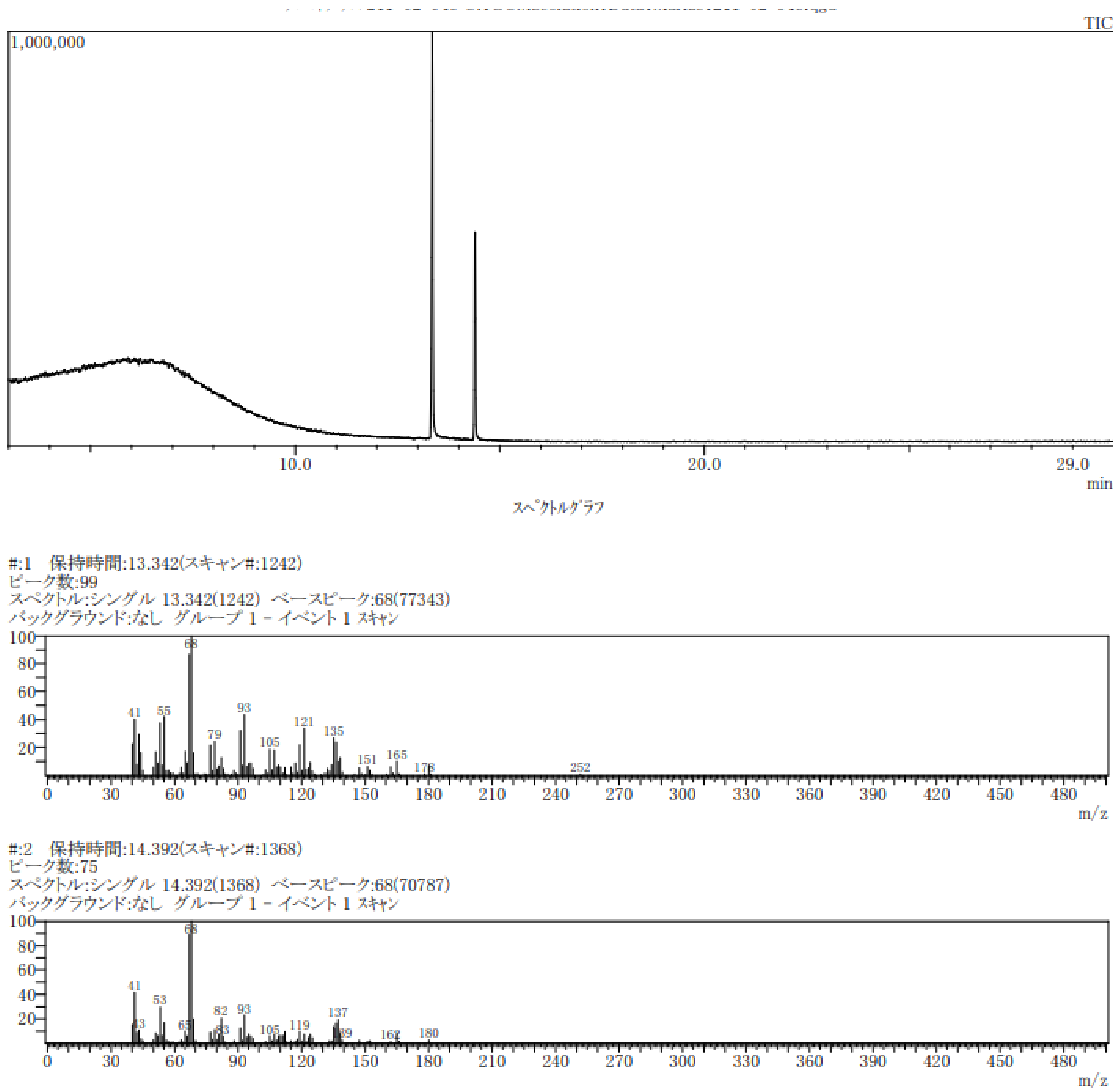


**Figure S22.** GC-MS chromatogram of monomer **1**.

## 3.4 SEC, TG and DSC charts

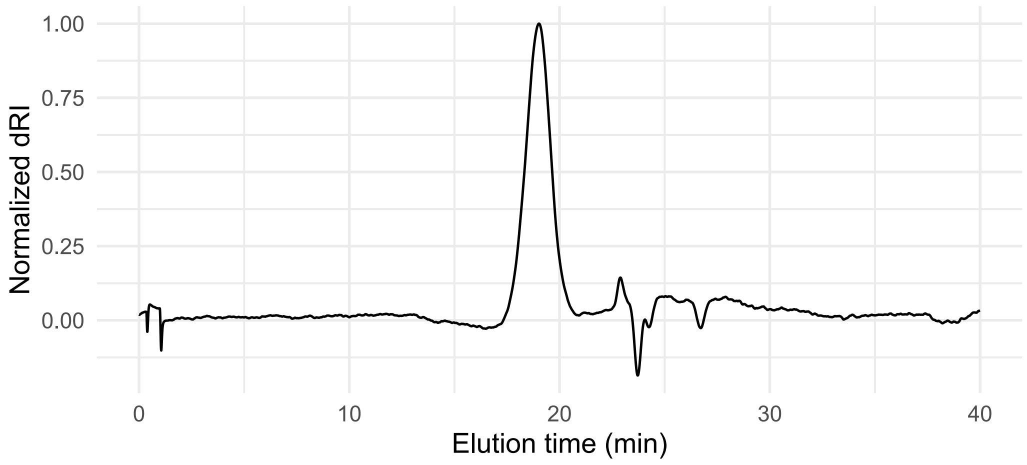


**Figure S23.** SEC charts of the $CO_2$/isoprene copolymer **2**.

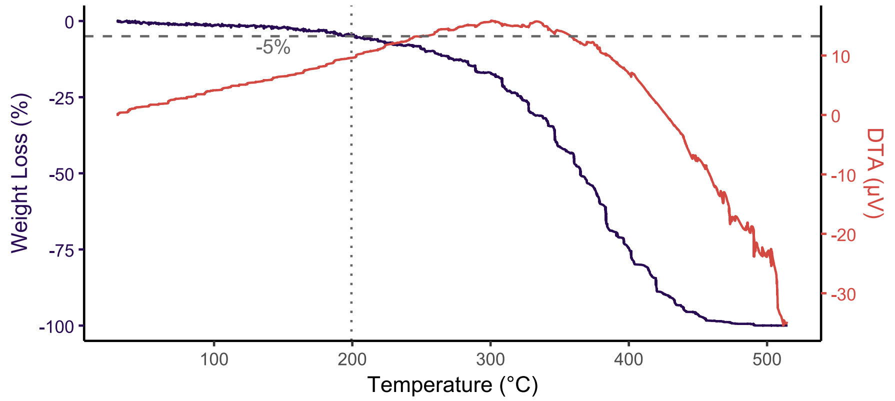


**Figure S24.** TG chart of $CO_2$/isoprene copolymer **2** measured at a heating rate of 10 °C/min from 30 °C to 515 °C. The y-axis represents normalized weight loss (black), and differential thermal analysis (DTA) (red).

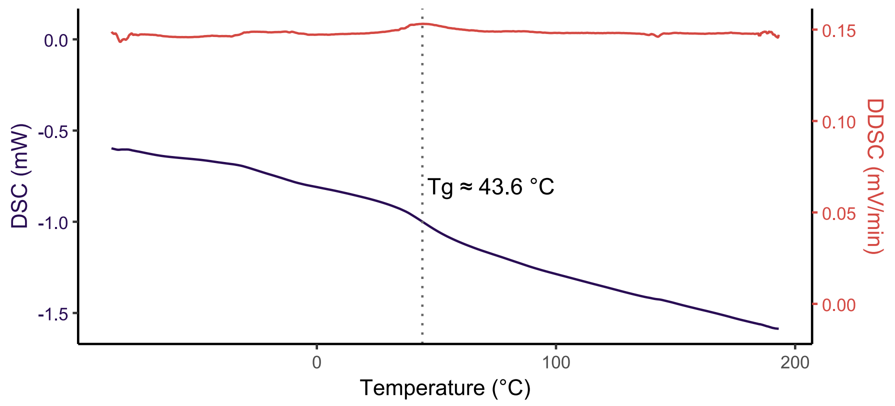


**Figure S25**. DSC chart of $CO_2$/isoprene copolymer **2**.

# 4. Supplementary References


(1) Reetz, M. T.; Maase, M. Redox-Controlled Size-Selective Fabrication of Nanostructured Transition Metal Colloids. *Adv. Mater.* **1999**, *11* (9), 773–777. https://doi.org/10.1002/(SICI)1521-4095(199906)11:9<773::AID-ADMA773>3.0.CO;2-1.

(2) Nagae, H.; Aoki, R.; Akutagawa, S.; Kleemann, J.; Tagawa, R.; Schindler, T.; Choi, G.; Spaniol, T. P.; Tsurugi, H.; Okuda, J.; Mashima, K. Lanthanide Complexes Supported by a Trizinc Crown Ether as Catalysts for Alternating Copolymerization of Epoxide and $CO_2$: Telomerization Controlled by Carboxylate Anions. *Angew. Chem. Int. Ed.* **2018**, *57* (9), 2492–2496. https://doi.org/10.1002/anie.201709218.

(3) Bieniek, J. C.; Mashtakov, B.; Schollmeyer, D.; Waldvogel, S. R. Dehydrogenative Electrochemical Synthesis of *N*-Aryl-3,4-Dihydroquinolin-2-ones by Iodine(III)-Mediated Coupling Reaction. *Chem. – Eur. J.* **2024**, *30* (7), e202303388. https://doi.org/10.1002/chem.202303388.

(4) Wei, C. S.; Davies, G. H. M.; Soltani, O.; Albrecht, J.; Gao, Q.; Pathirana, C.; Hsiao, Y.; Tummala, S.; Eastgate, M. D. The Impact of Palladium(II) Reduction Pathways on the Structure and Activity of Palladium(0) Catalysts. *Angew. Chem. Int. Ed.* **2013**, *52* (22), 5822–5826. https://doi.org/10.1002/anie.201210252.

(5) Song, J.; Feng, X.; Yamamoto, Y.; Almansour, A. I.; Arumugam, N.; Kumar, R. S.; Bao, M. Selective Synthesis of δ-Lactone via Palladium Nanoparticles-Catalyzed Telomerization of CO2 with 1,3-Butadiene. *Tetrahedron Lett.* **2016**, *57* (29), 3163–3166. https://doi.org/10.1016/j.tetlet.2016.06.024.

(6) Wang, J.; Feng, X.; Song, J.; Gao, Z.; Wang, W.-H.; Bao, M. The Ultrasmall Palladium Nanoparticles Catalyzed Telomerization of CO2 with 1,3-Butadiene at Room Temperature: Selective Synthesis of δ-Lactone. *ChemistrySelect* **2020**, *5* (30), 9404–9408. https://doi.org/10.1002/slct.202002203.

(7) Singh, S. P.; Iwasaki, T.; Terao, J.; Kambe, N. Cross-Coupling of Grignard Reagents with Alkyl Halides or Tosylates by the Use of Nickel or Palladium Containing Perovskite. *Tetrahedron Lett.* **2011**, *52* (7), 774–776. https://doi.org/10.1016/j.tetlet.2010.12.011.

(8) Nakano, R.; Ito, S.; Nozaki, K. Copolymerization of Carbon Dioxide and Butadiene via a Lactone Intermediate. *Nat. Chem.* **2014**, *6* (4), 325–331. https://doi.org/10.1038/nchem.1882.

(9) Liu, M.; Sun, Y.; Liang, Y.; Lin, B.-L. Highly Efficient Synthesis of Functionalizable Polymers from a $CO_2$/1,3-Butadiene-Derived Lactone. *ACS Macro Lett.* **2017**, *6* (12), 1373–1378. https://doi.org/10.1021/acsmacrolett.7b00774.

(10) Tang, S.; Zhao, Y.; Nozaki, K. Accessing Divergent Main-Chain-Functionalized Polyethylenes via Copolymerization of Ethylene with a $CO_2$/Butadiene-Derived Lactone. *J. Am. Chem. Soc.* **2021**, *143*, 17953–17957. https://doi.org/10.1021/jacs.1c08578.

(11) Hill, M. R.; Tang, S.; Masada, K.; Hirooka, Y.; Nozaki, K. Incorporation of CO2-Derived Bicyclic Lactone into Conventional Vinyl Polymers. *Macromolecules* **2022**. https://doi.org/10.1021/acs.macromol.1c02503.

(12) Garcia Espinosa, L. D.; Williams-Pavlantos, K.; Turney, K. M.; Wesdemiotis, C.; Eagan, J. M. Degradable Polymer Structures from Carbon Dioxide and Butadiene. *ACS Macro Lett.* **2021**, *10* (10), 1254–1259. https://doi.org/10.1021/acsmacrolett.1c00523.

(13) Piva, O. Direct Conversion of β,γ-Unsaturated Esters into Lactones Induced by TMS-I. *Tetrahedron* **1994**, *50* (48), 13687–13696. https://doi.org/10.1016/S0040-4020(01)85681-7.

(14) Hensinger, M. J.; Dodge, N. J.; Brewer, M. Substituted α-Alkylidene Cyclopentenones via the Intramolecular Reaction of Vinyl Cations with Alkenes. *Org. Lett.* **2019**. https://doi.org/10.1021/acs.orglett.9b04255.

(15) Iio, K.; Kobayashi, K.; Matsunaga, M. Radical Polymerization of Allyl Alcohol and Allyl Acetate. *Polym. Adv. Technol.* **2007**, *18* (12), 953–958. https://doi.org/10.1002/pat.870.

(16) Pracht, P.; Bohle, F.; Grimme, S. Automated Exploration of the Low-Energy Chemical Space with Fast Quantum Chemical Methods. *Phys. Chem. Chem. Phys.* **2020**, *22* (14), 7169–7192. https://doi.org/10.1039/C9CP06869D.

(17) Frisch, M. J.; Trucks, G. W. Gaussian 16, Revision C.01, 2016.

(18) Bootsma, A.; N.; Wheeler, S. Popular Integration Grids Can Result in Large Errors in DFT-Computed Free Energies. *ChemRxiv* **2019**. https://doi.org/10.26434/CHEMRXIV.8864204.V5.

(19) Perdew, J. P.; Burke, K.; Ernzerhof, M. Generalized Gradient Approximation Made Simple. *Phys. Rev. Lett.* **1996**, *77* (18), 3865–3868. https://doi.org/10.1103/PhysRevLett.77.3865.

(20) Perdew, J. P.; Burke, K.; Ernzerhof, M. Generalized Gradient Approximation Made Simple. *Phys. Rev. Lett.* **1997**, *78* (7), 1396–1396. https://doi.org/10.1103/PhysRevLett.78.1396.

(21) Ernzerhof, M.; Scuseria, G. E. Assessment of the Perdew–Burke–Ernzerhof Exchange-Correlation Functional. *J. Chem. Phys.* **1999**, *110* (11), 5029–5036. https://doi.org/10.1063/1.478401.

(22) Grimme, S.; Antony, J.; Ehrlich, S.; Krieg, H. A Consistent and Accurate Ab Initio Parametrization of Density Functional Dispersion Correction (DFT-D) for the 94 Elements H-Pu. *J. Chem. Phys.* **2010**, *132* (15), 154104. https://doi.org/10.1063/1.3382344.

(23) Grimme, S.; Ehrlich, S.; Goerigk, L. Effect of the Damping Function in Dispersion Corrected Density Functional Theory. *J. Comput. Chem.* **2011**, *32* (7), 1456–1465. https://doi.org/10.1002/jcc.21759.

(24) Marenich, A. V.; Cramer, C. J.; Truhlar, D. G. Universal Solvation Model Based on Solute Electron Density and on a Continuum Model of the Solvent Defined by the Bulk Dielectric Constant and Atomic Surface Tensions. *J. Phys. Chem. B* **2009**, *113* (18), 6378–6396. https://doi.org/10.1021/jp810292n.

(25) Ribeiro, R. F.; Marenich, A. V.; Cramer, C. J.; Truhlar, D. G. Use of Solution-Phase Vibrational Frequencies in Continuum Models for the Free Energy of Solvation. *J. Phys. Chem. B* **2011**, *115* (49), 14556–14562. https://doi.org/10.1021/jp205508z.

(26) Grimme, S. Supramolecular Binding Thermodynamics by Dispersion-Corrected Density Functional Theory. *Chem. – Eur. J.* **2012**, *18* (32), 9955–9964. https://doi.org/10.1002/chem.201200497.

(27) Luchini, G.; Alegre-Requena, J. V.; Funes-Ardoiz, I.; Paton, R. S. GoodVibes: Automated Thermochemistry for Heterogeneous Computational Chemistry Data. *F1000Research* **2020**, *9*, 291. https://doi.org/10.12688/f1000research.22758.1.

(28) Legault, C. Y. CYLview20, 2020. http://www.cylview.org.

(29) Bogdos, M. K.; Morandi, B. EveRplot: A Web-Based Shiny Application for Creating Energy vs Reaction Coordinate Diagrams. *J. Chem. Educ.* **2023**, *100*, 3641–3644. https://doi.org/10.1021/acs.jchemed.3c00319.
