# Supplementary material for: Gram-scale selective telomerization of isoprene and CO2 toward 100% renewable materials
Source: Nat Commun. 2025 Aug 28;16:7326. doi: 10.1038/s41467-025-62409-2 (PMC12394455; doi:10.1038/s41467-025-62409-2)
Supplement: Supplementary file 2 — Description of Additional Supplementary Files [file 41467_2025_62409_MOESM2_ESM.pdf]

Description of Additional Supplementary Files

Supplementary Data 1: Cartesian coordinates of structures for DFT experiments
